# Vitamin B6 Pathway Maintains Glioblastoma Cell Survival in 3D Spheroid Cultures

**DOI:** 10.3390/ijms251910428

**Published:** 2024-09-27

**Authors:** Najla Yussuf Moosa, Sara Abdullah Azeem, John K. Lodge, William Cheung, Shafiq Uddin Ahmed

**Affiliations:** 1 School of Medicine, Murray Health, Faculty of Health Sciences and Wellbeing, University of Sunderland, Sunderland SR1 3SD, UK; najla.moosa@sunderland.ac.uk (N.Y.M.); bh22us@research.sunderland.ac.uk (S.A.A.); 2 School of Human Sciences, London Metropolitan University, Tower Building, Holloway Road, London N7 8DB, UK; j.lodge@londonmet.ac.uk; 3 Department of Applied Sciences, Northumbria University, Ellison Building, Northumberland Road, Newcastle Upon Tyne NE1 8ST, UK; william.cheung@northumbria.ac.uk

**Keywords:** glioblastoma, vitamin B6, metabolomics, metabolic pathways, 3D culture

## Abstract

Glioblastoma (GBM) is a deadly brain cancer. The prognosis of GBM patients has marginally improved over the last three decades. The response of GBMs to initial treatment is inevitably followed by relapse. Thus, there is an urgent need to identify and develop new therapeutics to target this cancer and improve both patient outcomes and long-term survival. Metabolic reprogramming is considered one of the hallmarks of cancers. However, cell-based studies fail to accurately recapitulate the in vivo tumour microenvironment that influences metabolic signalling and rewiring. Against this backdrop, we conducted global, untargeted metabolomics analysis of the G7 and R24 GBM 2D monolayers and 3D spheroid cultures under identical cell culture conditions. Our studies revealed that the levels of multiple metabolites associated with the vitamin B6 pathway were significantly altered in 3D spheroids compared to the 2D monolayer cultures. Importantly, we show that pharmacological intervention with hydralazine, a small molecule that reduces vitamin B6 levels, resulted in the cell death of 3D GBM spheroid cultures. Thus, our study shows that inhibition of the vitamin B6 pathway is a novel therapeutic strategy for the development of targeted therapies in GBMs.

## 1. Introduction

Glioblastoma (GBM) is the most common form of primary brain tumour in adults. The World Health Organisation (WHO) classifies GBMs as grade IV astrocytomas. In the absence of treatment, GBM patients survive on average for less than 6 months. However, with an optimum treatment plan consisting of surgery, radiotherapy and concomitant temozolomide chemotherapy, median patient survival is 14–16 months [1]. Nevertheless, GBM remains an incurable cancer, marked by resistance to treatment modalities and poor prognosis. Tumour recurrence is inevitable, and this is thought to be driven by inter-and-intra-tumoral heterogeneity characterised by a distinct cell population with unique molecular and genetic characteristics [2,3]. Indeed, increasing evidence supports the notion that a small population of glioblastoma stem-like cells (GSCs) are resistant to conventional treatment and give rise to new tumours [4,5]. Thus, identification of small molecules that can target GSC population is likely to improve patient outcomes and long-term survival.

Metabolic reprogramming is considered one of the hallmarks of cancers [6]. Alteration of metabolic pathways is critical for maintaining high energy demands and metabolites necessary to support rapid tumour growth and proliferation [7,8,9]. This is particularly important in GBMs, which exhibit hypoxic and nutrient stress driven by poor tumour vasculature. In gliomas, mutations in isocitrate dehydrogenase 1 and 2 (IDH1/2) are known to lead to the production of the oncometabolite 2-hydroxyglutaric acid (2-HG) [10,11]. 2-HG is thought to increase the expression of hypoxia-induced factor-1α (HIF-1α) leading to upregulation of glucose transporter 1 (Glut 1) and hexokinase 2 (HK 2), as well as broader regulation of pathways associated with glutamine, fatty acids, and citrate biosynthesis [12,13,14,15,16]. Indeed, the difference in glucose uptake between the normal cells and the tumour cells is exploited clinically with ^18^F-Fluoro-deoxy-glucose positron emission tomography (^18^F-FDG PET) and with increased uptake of glucose shown to correlate with poor prognosis in GBMs [17,18,19].

Alteration in amino acid metabolism supports biomass production, ATP generation, and redox homeostasis in tumours [20,21,22]. Metabolomic studies have shown that glutamate levels are higher in glioma compared to normal brain tissue [23], and we have shown that glutamate-derived glutamine is able to satisfy the glutamine needs of GBMs, indicating that glutamine anaplerosis is dispensable for tumour growth [24]. Furthermore, changes in metabolic signatures have also been identified in gliomas according to tumour grades, with higher levels of arginine observed in higher grade gliomas compared to lower grade gliomas [25]. In contrast, defects in the arginine biosynthetic pathway due to epigenetic silencing of argininosuccinate synthetase (ASS1) has been associated with poor prognosis in GBM, indicating that metabolic circuitries are subject to complex molecular and genetic regulations [26].

Adherent 2D GBM cell cultures are widely used as convenient models for biological studies [27,28,29]. However, these monolayer cell cultures fail to accurately recapitulate in the vivo tumour microenvironment. In contrast, 3D GBM spheroid cultures are well-established, and have been shown to exhibit hallmarks of in vivo tumours, including hypoxic and nutrient gradients, a complex cellular phenotype with distinct zones of cellular proliferation, and areas of necrosis [30,31,32,33,34]. Such contrasting differences in cell culture models are likely to impinge on molecular and cellular signalling, thereby altering metabolic pathways. Against this backdrop, we conducted an unbiased global metabolomic analysis in 2D and 3D GBM cultures under identical cell culture mediums. Our studies reveal a distinct alteration of metabolic pathways in 3D cultures and identify vitamin B6 pathway as a novel regulatory mechanism governing GBM cell survival.

## 2. Results

### 2.1. Characterisation of Cells under 2D and 3D Cell Culture Conditions

**Figure 1:**
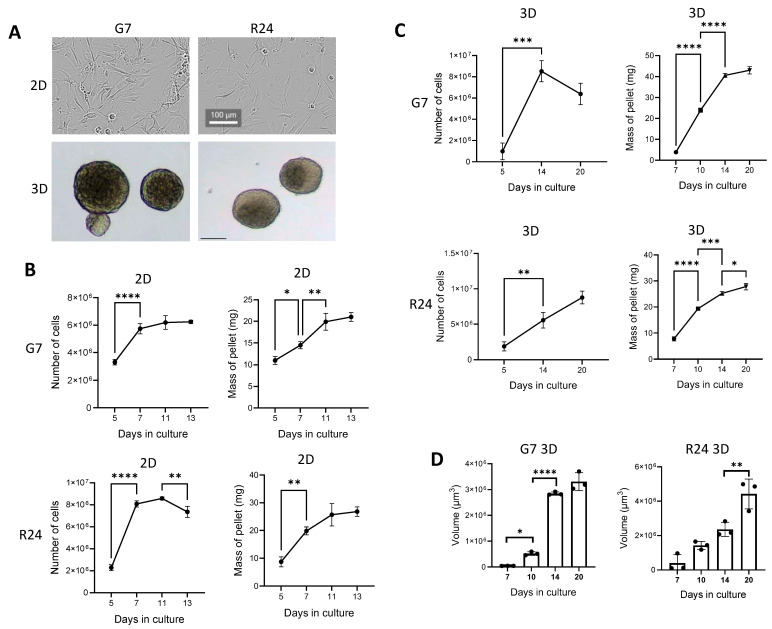
Analysis of cell proliferation in 2D and 3D GBM cultures. (**A**) Representative microscopic images of G7 and R24 2D monolayer cultures on matrigel and 3D spheroid cultures in suspension. Scale bar = 100 μm. Analysis of G7 and R24 (**B**) 2D and (**C**) 3D cell proliferation in T75 flasks and the corresponding wet cell pellet mass at various timepoints. (**D**) Bar chart shows mean spheroid volume in G7 and R24 spheroid cultures over a 20 day growth periods. Graphs show mean ± SD (*n* = 3), one way ANOVA and *t*-test, * *p* < 0.05, ** *p* < 0.005, *** *p* < 0.0005, **** *p* < 0.00005.

G7 and R24 GBM cell lines were cultured as a 2D adherent monolayer on matrigel or as 3D spheroids in suspension in T75 flasks, under identical cell culture media known to enrich and maintain the Glioblastoma stem cell (GSC) population [5] (Figure 1A). Proliferation of both G7 and R24 2D cell cultures reached a peak on day 7, and then plateaued (Figure 1B). However, the wet cell pellet mass continued to increase up to day 11, highlighting potential changes in cellular composition rather than cell number (Figure 1B). Corresponding G7 and R24 3D spheroid cultures exhibited steady increases in cell proliferation up to day 14, marked by increases in cell number, wet cell pellet mass and spheroid volume (Figure 1C,D). Interestingly, spheroids in 3D cultures were heterogenous, and this was particularly evident on day 14 and 21 (Supplementary Figure S1).

### 2.2. Validation and Molecular Analysis of 2D and 3D Spheroid Cultures

**Figure 2:**
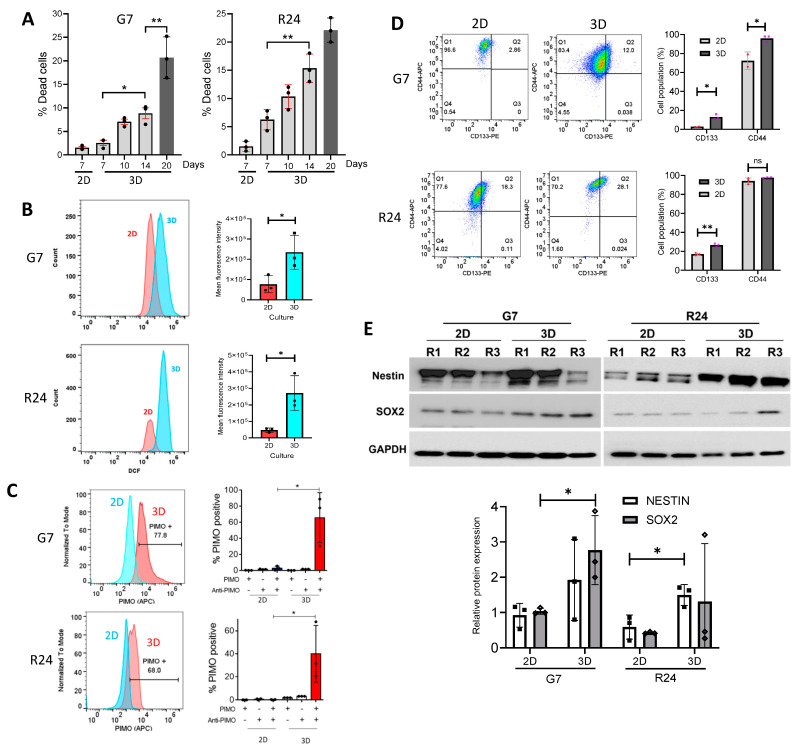
Biological characterisation of 2D and 3D GBM cell cultures. (**A**) Bar charts summarising flow cytometric data analysing the percentage of 7AAD positive cell population in 2D cultures on day 7 and 3D cultures over a period of 20 days in G7 and R24 GBM cell lines. (**B**) Representative flow cytometry overlay plots showing basal levels of reactive oxygen species in 2D and 3D GBM cultures on day 7 and 14 respectively. Bar charts summarising mean DCF fluorescence intensity in 2D and 3D cultures. (**C**) Representative flow cytometry overlay plots showing 2D and 3D cultures on day 7 and 14 respectively, following treatment with PIMO for 72 h. Bar charts summarise the percentage of PIMO positive hypoxic cells in the two cultures in the presence or absence of PIMO in combination with control or anti-PIMO antibody. (**D**) Representative flow cytometry plots and bar charts summarising the percentage of CD133 and CD44 positive cells in G7 and R24 2D and 3D cultures on day 7 and 14 respectively. (**E**) Representative Western blots showing the levels of Nestin and SOX2 stem cell markers in 2D and 3D G7 and R24 cultures on day 7 and 14 respectively in three independent biological replicates (R1–R3). Bar charts show densitometry analysis of Nestin and SOX2 normalised to GAPDH loading control. Bar charts show mean ± SD (*n* = 3), one way ANOVA and unpaired *t*-test, * *p* < 0.05, ** *p* < 0.005, ns = non-significant.

Two-dimensional and three-dimensional cultures exhibit distinct morphological differences, which can impinge on cell viability. Since our cell proliferation studies showed optimum cell growth on day 7 in 2D cultures and day 14 in 3D spheroid cultures (Figure 1), we aimed to examine how cell viability was impacted at these specific time points in both 2D and 3D environments. Flow cytometric analysis in G7 and R24 spheroid cultures on day 20 highlighted a time-dependent induction in dead cell population, reaching a peak of approximately 22% and 23%, respectively (Figure 2A). This contrasts with G7 and R24 2D cultures, which exhibited approximately 2% dead cell population on day 7 (Figure 2A). Oxidative stress has been shown to contribute to cellular stress and cell death in 3D cultures [35]. Analysis of reactive oxygen species (ROS) using 2′,7′-dichlorofluorescein (DCF) revealed significantly higher levels of ROS in 3D cultures compared to 2D cultures (Figure 2B). This could account for the increased cell death population in 3D spheroid cultures (Figure 2A). Cells in 3D cultures have also been shown to reside in a hypoxic microenvironment. To investigate this, the small molecule 2-nitroimidazole, (pimonidazole; PIMO) was used to selectively quantify hypoxic cells. Analysis of PIMO positive cells revealed that approximately 60% and 40% of the cell in the G7 and R24 spheroid, respectively, were hypoxic on day 14 compared to less than 1% in the corresponding 2D cultures (Figure 2C). Furthermore, flow cytometric analysis of membrane Glioblastoma stem cell markers (GSC) revealed that GBM cells express higher levels of CD44 compared to CD133 (Figure 2D). However, the expression of CD133 and CD44 was significantly higher in 3D G7 cultures compared to 2D cultures, whereas only CD133 levels were significantly higher in R24 3D spheroid cultures (Figure 2D). Western blot analysis of internal GCS markers revealed significantly higher levels of SOX2 in G7 and Nestin in R24 3D cultures compared to the corresponding 2D cultures (Figure 2E). Interestingly, passage-specific differences in the levels of SOX2 and Nestin was observed under each culture condition. Taken together, our data showed that 3D spheroid cultures exhibited higher levels of stem cell markers and displayed phenotypic changes associated with the in vivo GBM tumour microenvironment.

### 2.3. Metabolomic Analysis of 2D Monolayer and 3D Spheroid Cultures

**Figure 3:**
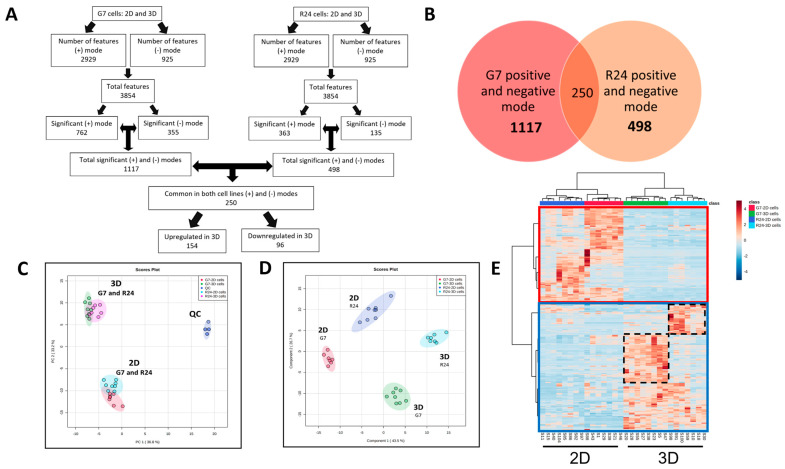
Metabolomic analysis of 2D and 3D G7 and R24 cultures. (**A**) Flow chart summarising the number of features identified at various stages of the LC-MS based metabolomics data. analysis pipeline in positive (+) and negative (−) ionisation modes in G7 and R24 cells resulting in the identification of (**B**) 250 features that were significantly altered between 2D and 3D cultures in both cell lines (*p* < 0.05, FDR < 0.046 and VIP > 1.1). (**C**) Principal component analysis (PCA) highlighting grouping between 2D and 3D samples and (**D**) Partial Least Squares Discriminant Analysis (PLS-DA) score plot highlighting clear separation between 2D, 3D and quality control (QC) samples in the G7 and R24 cell lines. (**E**) Heatmap with hierarchical clustering highlighting the 250 significantly altered features including 154 that were significantly higher (blue box) and 96 that were significantly lower (red box) in 3D cultures compared to 2D. Cell line specific clusters are highlighted in black dashed boxes.

Untargeted global metabolomic analysis was performed in the R24 and G7 2D monolayer and 3D spheroid cultures under optimum growth conditions on day 7 and 14, respectively. Up to eight independent biological replicates were generated under 2D and 3D cell culture conditions. All samples were processed and analysed simultaneously using ultra-high performance liquid chromatography-quadrupole time-of-flight mass spectrometry (UHPLC-QTOF MS). A total of 3854 features were identified in R24 and G7 cultures under positive and negative ionisation modes (Figure 3A). Analysis of the features using the MetaboAnalyst software (www.metaboanalyst.ca, Version 4) revealed 1117 and 498 features that were significantly altered between 2D and 3D cultures in G7 and R24 cell lines, respectively. Importantly, combined analysis of G7 and R24 cultures highlighted 250 features that were significantly altered between 2D and 3D cultures in both cell lines (Figure 3B). This included 154 up- and 96 downregulated features in 3D compared to 2D cultures. A summary of all 250 features, including fold change (FC), log_2_FC, *p*-value, VIP score, and false discovery rate (FDR) is presented in Supplementary Table S1.

### 2.4. Multivariate Analysis of Significantly Altered Features in 2D vs. 3D GBM Cultures

Multivariate analysis of the 250 significantly altered features in the two GBM cell lines highlighted notable metabolic differences between the 2D monolayer and 3D spheroid cultures. Principal component analysis (PCA) highlighted three distinct clusters consisting of quality control (QC), 2D and 3D samples (Figure 3C). Supervised partial least squares discriminant analysis (PLS-DA) resulted in maximum separation between 2D and 3D cell culture models, resulting in four distinct clusters consisting of G7 and R24, 2D or 3D samples (Figure 3D). Component 1 in the PLS-DA plot captured the different cell culture conditions, whereas component 2 highlighted differences between the G7 and R24 cell lines. A heatmap of the 250 significantly altered features between 2D and 3D cultures across both cell lines was generated using the Ward clustering algorithm on the MetaboAnalyst software package (Figure 3E). Overall, there was a distinct separation of the 154 up- and 96 downregulated features in 3D compared to 2D cultures. However, there were some features in ‘smaller clusters’ which were upregulated to a greater magnitude in 3D cultures in a cell-line-specific manner (Figure 3E, dashed boxes).

### 2.5. Identification and Validation of Significantly Altered Pathways in GBM 3D Spheroid Cultures

The 250 significantly altered features in 3D spheroid cultures were subject to putative metabolite identification using the Human Metabolome Database (www.hmdb.ca) using the accurate *m*/*z* values with a tolerance set at 20 parts per million (ppm). This resulted in the identification of 287 and 107 putative metabolites associated with the 154 up and 96 downregulated features in 3D spheroid cultures (Supplementary Tables S2 and S3).

**Figure 4:**
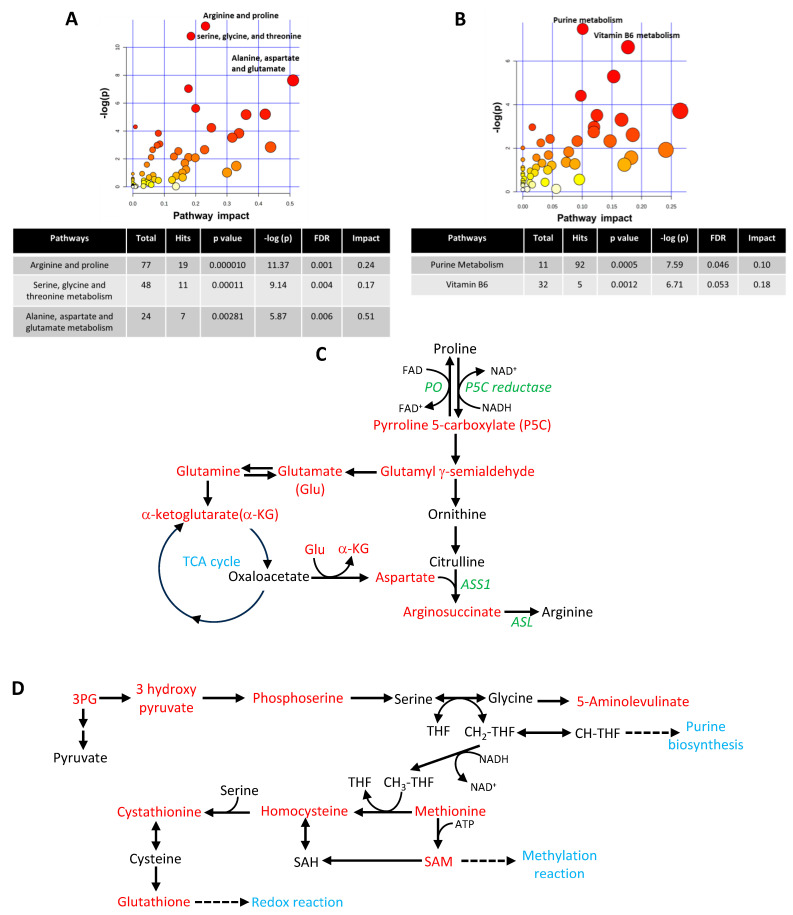
Analysis of significantly altered pathways in 3D cultures. Metabolome view highlighting putative metabolites contributing to metabolic pathways that are significantly (**A**) upregulated and (**B**) downregulated in G7 and R24 3D cultures compared to 2D. Size of the circle corresponds to the number of metabolites contributing towards a specific pathway (pathway impact) and statistical significance is highlighted by the color (red to yellow). The three most significantly up and two most significantly downregulated pathways in 3D cultures have been highlighted on the plots and summarised in the tables. Analysis was performed on MetaboAnalyst using the pathway impact module. (**C**) Schematic linking key putative metabolites associated with proline, arginine and glutamine pathways that were significantly higher in 3D cultures compared to 2D (red). Proline and arginine metabolism is closely linked with the Tricarboxylic Acid (TCA) cycle. Key enzymes involved in the metabolic steps are shown in green. (**D**) Putative metabolites associated with serine and glycine metabolism pathways that were significantly higher in 3D compared to 2D (red). Serine can be formed de novo from glycolysis through the oxidation of the metabolic intermediate 3-phosphoglycerate (3-PG). Serine is a precursor for the folate cycle and one-carbon metabolism, purine biosynthesis and provides intermediates for methylation reactions. The folate cycle is also linked to the methionine biosynthesis, which is involved in the production of glutathione for redox reactions. Abbreviations: CH-THF—5,10-methylenetetrahydrofolate; THF—tetrahydrofolate; SAM—S-adenosylmethionine; SAH—S-Adenosyl Homocysteine; PO—Proline oxidase; ASS1—Arginosuccinate synthetase 1; ASL—Arginosuccinate lyase.

The putative up- and downregulated metabolites were uploaded separately onto the MetaboAnalyst platform and interrogated using the Metabolic Pathway Analysis (MetPA) module. This resulted in the identification of four pathways that were significantly upregulated and two pathways that were significantly downregulated in 3D cultures (*p* < 0.05 and FDR ≤ 0.05) (Supplementary Tables S4 and S5). The top three significantly upregulated pathways in 3D cultures were: (i) arginine and proline, (ii) serine, glycine, and threonine, and (iii) alanine, aspartate, and glutamate metabolism pathways (Figure 4A). Key putative metabolites associated with the significantly upregulated pathways have been highlighted (Figure 4C,D), and the full list has been provided in Supplementary Table S6.

**Figure 5:**
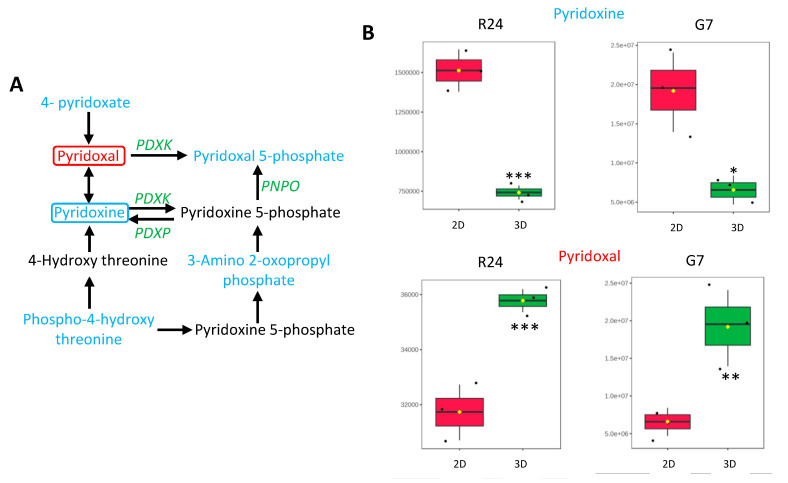
Vitamin B6 pathway is significantly altered in 3D spheroid cultures. (**A**) Schematic showing putative metabolites associated with vitamin B6 pathway that were significantly lower in 3D cultures compared to 2D (blue). Enzymes associated with key steps in vitamin B6 synthesis is shown in green (**B**) Box plots generated from LC-MS validation studies showing concentration (signal intensity) of pyridoxal and pyridoxine in R24 and G7 2D and 3D cultures. Levels of pyridoxine were significantly lower, whereas levels of pyridoxal were significant higher in 3D cultures compared to 2D. The black dots represent the concentration of metabolite associated with each sample (*n* = 3), mean concentration is highlighted by yellow diamond, unpaired *t*-test, * *p* < 0.05, ** *p* < 0.005, *** *p* < 0.0005. Abbreviations: PDXK—Pyridoxal kinase; PDXP—Pyridoxal phosphate phosphatase; PNPO—Pyridoxamine 5′-Phosphate oxidase.

Purine and vitamin B6 metabolism pathways were the two most significantly downregulated pathways in 3D spheroid cultures compared to 2D cultures (*p* < 0.001, and FDR ≤ 0.05) (Figure 4B). List of the putative metabolites associated with the purine pathway have been summarised in Supplementary Table S7. In the vitamin B6 pathway, 4-pyridoxate, pyridoxal 5-phosphate, 3-amino 2-oxopropyl phosphate, phospho-4-hydroxy threonine and pyridoxal/pyridoxine were identified as significantly lower in 3D spheroid cultures compared to 2D monolayer cultures (Figure 5A). Since little is known about the role of vitamins in GBMs, changes in metabolites associated with the vitamin B6 pathway in 3D cultures were investigated further. For this purpose, metabolic analysis was performed to validate changes in the levels of pyridoxal and pyridoxine, two of the key forms of vitamin B6 in 2D and 3D GBM cell cultures. UHPLC-QTOF MS data revealed significantly lower levels of pyridoxine in G7 and R24 3D cultures compared to 2D cultures (Figure 5B). Interestingly, the levels of pyridoxal were significantly higher in 3D compared to 2D cultures. Such contrasting differences between the two different forms of vitamin B6 could be due to the complex metabolic regulatory feedback loop governing the cellular levels of pyridoxal, pyridoxine, and their active phosphorylated forms in cells (Figure 5A).

### 2.6. Targeting the Vitamin B6 Pathway in GBMs

**Figure 6:**
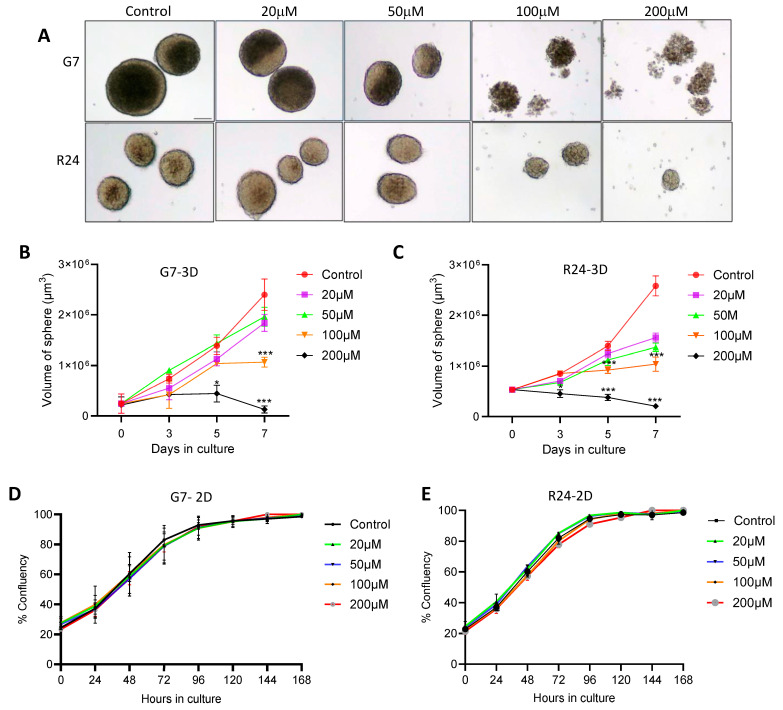
Inhibition of vitamin B6 pathway preferentially effects GBM 3D spheroids. (**A**) Representative microscopic images of R24 and G7 3D spheroids treated on day 7 with 20, 50, 100, and 200 μM hydralazine and then incubated for 7 days. Images taken at 4× magnification. Scale bar = 100 μm. Graphs summering mean spheroid volume in (**B**) G7 and (**C**) R24 3D spheroids cultures over the 7 days hydralazine treatment. 35–40 spheroids analysed per time point, *n* = 3 independent experiment, mean ± SD shown. Graphs showing the percentage cell confluency in (**D**) G7 and (**E**) R24 2D cell lines cultured for 24 h and then subject to hydralazine treatment at various concentrations over 168 h, *n* = 10 wells per experiment, three independent experiments, mean ± SD shown. Two-way ANOVA, and *t*-test vs control, * *p* < 0.05, *** *p* < 0.0005.

We hypothesised that the vitamin B6 pathway could play a crucial role in the maintenance of cells in 3D spheroid cultures. To test this, we utilised the small molecule calcium transport inhibitor hydralazine, which is known to bind and enhance vitamin B6 excretion in cells [36,37]. G7 and R24 GBM spheroids were cultured for 7 days and then treated with hydralazine at a range of concentrations. Images of the spheroids were taken to calculate the volumes on days 3, 5, and 7. Treatment of G7 3D cultures with 100 μM and 200 μM hydralazine for 7 days resulted in significant reduction of spheroid volume compared to control treatment (Figure 6B). Interestingly, R24 cultures were more sensitive to hydralazine compared to G7 cultures, exhibiting a dose-dependent reduction in spheroid volume at all treatment concentrations on day 7 (Figure 6C). Importantly, we observed a dose-dependent disintegration of the spheroid cultures in response to hydralazine treatment resulting in floating single cells visible under the microscope (Figure 6A). To determine if the effects of hydralazine were specific to 3D cultures, corresponding G7 and R24 2D monolayer cultures were treated with hydralazine for 7 days. Strikingly, 2D cell proliferation remained unaffected at all concentrations for the duration of hydralazine treatment (Figure 6D,E), underscoring that the growth inhibitory effects of hydralazine were specific to GBM 3D spheroid cultures.

### 2.7. Mechanistic Studies Investigating the Effects of Hydralazine in 3D Spheroid Cultures

**Figure 7:**
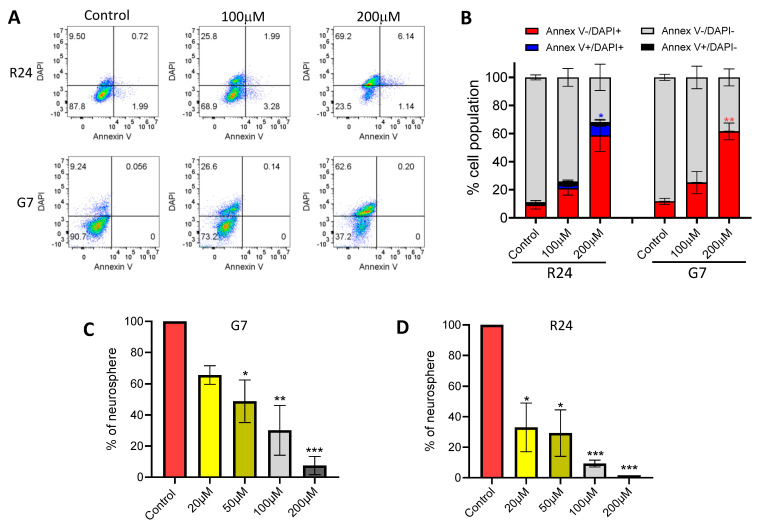
Inhibition of vitamin B6 pathway leads to loss of cell viability and neurosphere formation. (**A**) Representative flow cytometric plots showing percentage of annexin V and/or DAPI cell population in G7 and R24 3D GBM spheroids cultured for 7 days and then treated with 100 μM and 200 μM of hydralazine for 4 days. (**B**) Quantification of annexin V and DAPI cell populations. Red and blue bars highlight non-apoptotic DAPI+ and late apoptotic annexin V and DAPI+ cell populations, respectively. Mean ± SD shown, *n* = 3 independent experiment. Bar charts showing the percentage of neurosphere formation in (**C**) G7 and (**D**) R24 cell lines. Number of neurospheres were counted following treatment of single cells with various concentrations of hydralazine for 28 days. 10 wells per experiment, mean ± SD shown, *n* = 3 independent experiments. One way ANOVA and *t*-test vs control * *p* < 0.05, ** *p* < 0.005, *** *p* < 0.0005.

To investigate the potential mechanism governing the loss of cell viability in response to hydralazine, G7 and R24 spheroids were cultured for 7 days and then treated with hydralazine for 4 days. Flow cytometric analysis of combined annexin V and DAPI revealed significant dose-dependent increases in the percentage of DAPI+ cells in R24 and G7 spheroid cultures (Figure 7A,B). Indeed, treatment with 200 μM hydralazine resulted in up to 60% DAPI+ cells in GBM spheroid cultures, indicating a non-apoptotic mechanism as the primary diver of cell death in response to hydralazine treatment (Figure 7A,B). Interestingly, R24 spheroid cultures exhibited a small but significant dose-dependent increase in annexin V+ cell population, reaching a peak of approximately 10% in response to treatment with 200 μM hydralazine. Therefore, multiple cell death mechanisms regulate hydralazine-induced cell death in 3D spheroid cultures, which may operate in a cell line specific manner.

Since GBM stem cells (GSCs) are more resistant to treatment modalities, the effects of hydralazine on the ability of GSCs to form neurospheres was investigated. For this purpose, R24 and G7 cells were seeded into 96-well plates and treated with hydralazine at various concentrations. Analysis of neurospheres on day 21 revealed a significant dose-dependent reduction in the neurosphere formation capacity of G7 and R24 cells in response to hydralazine treatment (Figure 7C,D). Treatment with 200 μM hydralazine resulted in an approximately 90% reduction in G7 neurosphere formation, whereas an almost complete absence of neurospheres were observed in R24 cells. Indeed, R24 cells were more sensitive, exhibiting an approximately 70% reduction in neurosphere formation capacity following treatment with 20 μM hydralazine. Taken together, these results clearly show that hydralazine can effectively target the GCS population. 

## 3. Discussion

In this study, global metabolomics analysis of GBM cell cultures were performed under 2D monolayer and 3D spheroid conditions to identify unique metabolic pathways for therapeutic exploitation. The use of 3D spheroids is a well-established method for culturing GBM cells, providing a rapid, simple and superior model for investigating molecular and cellular processes compared to 2D monolayer cell cultures [27,28,30,31]. Our results show that 2D cultures were absent of hypoxic cells, whereas 3D spheroid cultures on day 14 consisted of 40–60% hypoxic cells, recapitulating one of the fundamental hallmarks of GBMs (Figure 2C). Indeed, hypoxia is thought to drive oxidative stress contributing to necrotic cell death, and increase GSC population in vivo [38,39]. This is consistent with our findings which show that 3D cultures on day 14 exhibited significantly higher levels of ROS and a 5–8-fold increase in dead cell population compared to the corresponding 2D cell culture (Figure 2A,B). Furthermore, the levels of Nestin, SOX2 and CD44 were also higher in 3D cultures in a cell-line-specific manner, highlighting that GBM spheroid cultures exhibit increased levels of stem cell markers compared to the corresponding 2D cultures.

An important prerequisite of metabolomic studies investigating cell culture models is the need to employ a standardised cell culture media and maintain key cellular phenotypes that recapitulate in the vivo tumour microenvironment. In this study, 2D and 3D GBM cells were cultured under identical cell culture media in the absence of FBS, which has been shown to deplete the GCS population [4,5]. Importantly, we conducted metabolomic studies in G7 and R24 2D and 3D cultures on day 7 and 14, respectively, which were based on optimum cell proliferation (Figure 1) and confirmation of hypoxia, ROS and acceptable level of cell death in 3D cultures (Figure 2). These parameters were crucial in ensuring that differences between 2D and 3D metabolomic profiles was not a consequence of external metabolic challenges [40]. 

An untargeted metabolomics approach was employed to identify differences between 2D and 3D GBM spheroid cultures. This ensured comprehensive, unbiased analysis of the metabolome and resulted in the identification of 3854 metabolic features in G7 and R24 GBM cell cultures. Crucially, combined analysis of both cell lines removed cell-line-specific differences and resulted in the identification of 250 features that were significantly altered between 2D and 3D cultures in G7 and R24 cell lines (Figure 3 and Supplementary Tables S1–S3).

The results presented in this study show that multiple closely connected putative metabolites associated with proline metabolism were significantly higher in the 3D spheroids compared to 2D cultures. These included higher levels of pyrroline-5-carboxylate (P5C), glutamate semialdehyde (GSA), glutamate (Glu), and α-ketoglutaric acid (α-KG) (Figure 4C and Supplementary Tables S4 and S6). Proline metabolism can be divided into two half cycles. In one half, catabolism of proline leads to P5C and downstream intermediates, and in the other half, P5C is converted to proline. Our data suggest that catabolism of proline in 3D spheroid cultures increases levels of proline catabolic half-cycle intermediates like P5C, GSA, Glutamate, and α-KG [41]. This finding is consistent with a previous study in breast cancer which shows that proline catabolism supports growth of 3D spheroids by increasing production of ATP through proline oxidase [42]. Furthermore, previous studies have shown that hypoxia can trigger metabolomic rewiring through proline catabolism, resulting in cancer cell survival [43]. In addition, oxidation of proline to P5C has been shown to produce reactive oxygen species (ROS), which could be a mechanism contributing to the increased levels of ROS observed in 3D GBM spheroid cultures [41].

Arginine is a conditionally essential amino acid produced using the intermediates citrulline and aspartate via a two-step reaction (Figure 4C and Supplementary Tables S4 and S7) [44]. In first step, citrulline and aspartate form argininosuccinic acid, which is catalysed by arginosuccinate synthetase (ASS1). In step two, arginosuccinate layse (ASL) converts argininosuccinic acid to arginine and fumarate. A previous study has shown that GBMs are typically deficient in either ASS1 or ASL, which hinders the production of arginine and subsequently leads to arginine deficiency [45]. However, the increased levels of aspartate and citrulline with concomitant increases in argininosuccinic acid in our data suggests a functional ASS1 in GBM 3D spheroid cultures. Furthermore, these findings are consistent with the recent publication showing that the arginine pathway is significantly upregulated in the GBM neurosphere and could be selectively targeted using the ASS1 inhibitor α-methyl-DL-aspartic acid (MDLA) [40]. However, in the absence of metabolic flux experiments, it remains unclear whether the unaltered levels of arginine observed in our 3D spheroid cultures was due to rapid dissemination of arginine into alternative metabolic pathways [46].

Serine and glycine are non-essential amino acids that play an important role in the production of proteins, nucleic acid and DNA [47]. One of the key pathways that serine feeds into is the folate cycle through the production of glycine. Previous studies have shown that serine–glycine metabolism is essential for tumorigenesis [48,49]. Interestingly, the levels of the putative metabolites 3-hydroxy pyruvate (3HP) and phosphoserine (PS) were higher in 3D spheroids compared to 2D cultures (Figure 4D and Supplementary Tables S4 and S6). This observation is consistent with alterations in the glycolytic flux under hypoxia, which leads to the generation of 3HP instead of pyruvate [50]. Interestingly, our data show that the levels of serine and glycine were not significantly altered in the 3D spheroid cultures. However, these two amino acids play essential role in the generation of one carbon units, which are crucial for the generation of macromolecules and maintenance of cellular redox. Indeed, in cancer cells, nucleotides and amino acids are required at a much higher rate to maintain rapid cell proliferation compared to normal cells. This is supported by data that show serine is critical for cancer cell proliferation [51]. 

The conversion of serine to glycine generates 5,10 methylenetetrahydrofolates (CH_2_-THF), which is a key intermediate in the folate cycle contributing to the biosynthesis of purine nucleotides. In addition, CH_2_-THF is also a key intermediate in the methionine cycle. Our data show that the levels of methionine, homocysteine, and SAM were all potentially higher in 3D spheroids compared to 2D cultures (Figure 4D and Supplementary Tables S4 and S6). In the methionine cycle, homocysteine is re-methylated using a one-carbon unit from CH_2_-THF to form methionine via methionine synthase (MS). Demethylation of S-adenosyl-methionine (SAM) yields S-adenosyl-homocysteine (SAH), which is then converted to homocysteine, completing the cycle. SAM is also a critical source of one carbon unit and participates in the methylation of proteins, DNA and RNA [52]. Previous studies have showed that methionine deprivation decreased tumoursphere formation and cancer cell stemness [53,54]. Indeed, our data shows that the expression of stem cell markers was overall higher in 3D GBM spheroid cultures compared to 2D cultures. Furthermore, homocysteine has been shown to play an important role in promoting and maintaining cancer stem cells, and contributes to the survival of aggressive tumours including glioblastomas [55,56,57].

The role of glutamine/glutamate in cancer cell metabolism is well established [58]. Glutamine provides anaplerotic carbons to supply the TCA cycle through α-ketoglutaric acid (α-KG), thus contributing to the production of ATP and the synthesis of intermediates for the generation of macromolecules [59]. Indeed, increased glutaminolysis correlates with carcinogenesis, and targeting these processes impairs cancer cell proliferation [60]. Our data suggests that glutamine, glutamate and α-KG were all significantly higher in 3D spheroids compared to 2D cultures (Figure 4C and Supplementary Tables S4 and S6). Glutaminase isoenzymes 1/2 (GLS-1 and GLS-2) function in the cytoplasm, catalysing the conversion of glutamine to glutamate. Levels of GLS have been shown to be pivotal in glutaminolysis and crucial for GBM cell survival and tumour growth [61]. Thus, targeting GLS is considered a therapeutic strategy in GBM [62,63]. Glutamate can be converted into α-ketoglutarate through cytoplasmic or mitochondrial transamination by amino acid-specific transaminases or via mitochondrial oxidative deamination by glutamate dehydrogenase 1 (GLDH1). In addition, transamination of glutamate also produces nonessential amino acids, such as aspartate, which was potentially significantly higher in 3D spheroids compared to 2D cultures. Aspartate plays a crucial role as a nitrogen donor in the de novo synthesis of purine and pyrimidines [64,65]. Interestingly, upregulation of nicotinamide metabolism and purine synthesis have been associated with maintenance of GSCs, and these pathways have been proposed as targets for drug development in GBM [66,67].

The significance of vitamins in cancer research is a developing field, with multiple studies suggesting that dietary vitamins and minerals may contribute to the prevention and treatment of variety of cancers including lungs, breast, liver and the brain [68]. However, this is in contrast to studies that have highlighted increased risk of various diseases, including cancers, associated with the long-term consumption of dietary supplements containing vitamins and minerals [69]. Using an unbiased metabolomics approach, we identified significantly lower level of multiple metabolites associated with the vitamin B6 pathway in 3D spheroid cultures (Figure 5 and Supplementary Tables S5 and S7). Vitamin B6 is crucial for numerous physiological functions and is associated with the synthesis of neurotransmitters, including serotonin, dopamine, and gamma-aminobutyric acid (GABA), which are essential for regulating normal neurological function and development [70]. Additionally, Vitamin B6 contributes to the integrity and functionality of myelin, which is essential for efficient transmission of electrical signals between neurons.

We confirmed that the levels of pyridoxine, a key form of Vitamin B6 was significantly lower in G7 and R24 3D spheroid compared to the 2D cultures. In contrast, pyridoxal levels were significantly higher in G7 and R24 3D spheroids, highlighting a reduction in pyridoxine/pyridoxal ratio. These opposing trends in the inactive forms of Vitamin B6 suggest an atypical utilization of metabolite in the Vitamin B6 pathway, potentially supporting the growth of GBM cells in the 3D spheroid microenvironment. The regulation of vitamin B6 is complex, involving intricate feedback mechanisms that are regulated by numerous enzyme and metabolic intermediates. Vitamin B6 exists in several forms, including pyridoxine, pyridoxal, and pyridoxamine, which all exist in the phosphorylated form, including the predominantly active pyridoxal 5′-phosphate (PLP) [70]. Indeed, our results showed that PLP levels were significantly lower in 3D cultures compared to 2D cultures. We speculated that this decrease in PLP, along with the elevated pyridoxal levels, could indicate rapid use of PLP by cells in 3D cultures. To investigate this further, we adopted a strategic approach to deplete vitamin B6 levels further by treating cells with hydralazine. Hydralazine is an antihypertensive medication which modulates the function of calcium and potassium channels as well as the nitric oxide (NO) pathway leading to vasodilation of vascular smooth muscles [71]. However, hydralazine is known to bind and enhance the excretion of vitamin B6 leading to the well documented Vitamin B6 deficiency in patients taking this drug [36,37].

Our results strikingly showed that treatment of G7 and R24 GBM 3D spheroid cultures with hydralazine resulted in a dose dependent decreased in spheroid volume. Treatment with 100 μM and 200 μM hydralazine resulted in disintegration of the spheroids, and flow cytometric analysis confirmed that up to 60% of cells were dead at the higher concentration. Flow cytometric analysis also confirmed that these cells were predominantly dying via a non-apoptotic mechanism. GSCs continue to be a pivotal focus of molecular therapeutics [72]. Our study demonstrates that hydralazine significantly inhibits neurosphere formation even at a 20 μM, suggesting the effects of hydralazine in part could be through targeting the GSC population. Crucially the effects of hydralazine was very specific to 3D spheroids as 2D cells were remarkably unaffected by the drug treatment, highlighting that the observed phenomena was not a consequence of general drug related cytotoxicity [73,74]. However, the exact mechanism of action of hydralazine in GBM is likely to be complex [75]. Previous studies have shown that hydralazine functions as a non-nucleoside DNA methyltransferase (DNMT) inhibitor leading to a global decrease in DNA methylation [73,74]. Indeed, various studies in prostate cancer cell lines have shown that hydralazine restored the expression of silence genes and functioned synergistically with the androgen receptor antagonist enzalutamide and the histone deacetylases inhibitor (HDACi) panobinostat to exert anti-tumour effects [76,77,78].

Vitamin B6 is a cofactor for over 150 enzymes, including those involved in synthesis and degradation of multiple amino acids, nucleic acids, as well as those controlling glucose, lipid and fatty acid metabolism [70]. Thus, oncogenic growth in GBM could be driven by metabolites associated with the vitamin B6 pathway. Indeed, this notion is supported by studies that show acute myeloid leukaemia (AML) is addicted to the vitamin B6 pathway, and genetic ablation of pyridoxal kinase (PDXK) results in the reduction of nucleic acids, polyamines and the activity of multiple PLP regulated enzymes, leading to cell death [79].

## 4. Materials and Methods

### 4.1. Glioblastoma Cell Culture

Primary GBM cell lines G7 and R24 were kindly gifted by Dr Colin Watts, University of Birmingham, UK, and have been previously characterised [4,5]. Tissue samples were obtained in accordance with local ethical guidelines. Cell culture was performed in a Class II biological safety cabinet using sterile plasticware and an aseptic technique. Two-dimensional and three-dimensional G7 and R24 cells were cultured in complete stem media consisting of advanced DMEM/F-12 (Gibco) supplemented with 0.66% B-27 (Gibco), 0.33% N-2 (Gibco), (ThermoFisher Scientific, Cramlington, UK), 0.1% heparin (Merck Life Science, Dorset, UK), 1% Glutamine (Gibco), 0.5% penicillin-streptomycin, 10 ng/mL fibroblast growth factor (Gibco) and 20 ng/mL epidermal growth factor (Gibco). Two-dimensional cells were cultured as monolayer on matrigel (Corning, Corning, NY, USA)-coated T75 flasks (1:60) and 3D spheroid cultures were maintained in suspension in matrigel free T75 flaks at 37 °C, 5% CO_2_ (Sanyo, Tokyo, Japan). Cells were routinely passaged at 70–80% confluency. Early passage cells were used throughout the studies, and cell cultures were discarded after passage 10.

### 4.2. 2D and 3D Cell Proliferation Studies

G7 and R24 cells were plated out on matrigel-coated T75 flasks at 1 × 10^5^ and 1.5 × 10^5^ cells respectively and harvested on day 5, 7, 11, and 13 to determine cell number and wet cell pellet mass. Three-dimensional spheroid cultures were generated by plating out 2.5 × 10^5^ and 7.5 × 10^5^ G7 and R24 cells in T75 flasks, respectively. Images of spheroid cultures were taken using an optical microscope (Lieca, Wetzlar, Germany) attached to a GXcam Hichrome camera (GT vision) at 4× or 10× magnification on day 7, 10, 14 and 20. Image J (NIH, Bethesda, MD, USA, Version 1.53i) was used to calculate the diameter of the spheroid, and the volume was calculated using: 4/3πr^3^. Three-dimensional flasks were also harvested at the same timepoints to determine the cell number and wet cell pellet mass.

### 4.3. Analysis of Cell Death and Apoptosis

Analysis of cell death was performed in 2D and 3D cell cultures using 7-aminoactinomycin D (7AAD) (Abcam, Cambridge, UK, ab142391). T75 flasks were harvested at specific timepoints, and the cell pellets were dissociated into single cell suspension and incubated with 400 µL cold PBS containing 0.2 μg/mL 7AAD for 15 min in the dark followed by flow cytometric analysis (BD C6 Accuri). Apoptotic cells were detected using the Annexin V apoptosis detection kit (BD, 559763) following the manufacturer’s protocol. Briefly, G7 and R24 3D spheroid cultures were generated as described earlier, and on day 7, treated with 100 μM and 200 μM of hydralazine (Sigma Aldrich, St. Louis, MO, USA, H1753-5G) for 48 h and harvested for flow cytometric analysis (Beckman CytoFLEX, Brea, CA, USA). All flow cytometry samples were filtered using a 70 µm filter (FlowmiTM), and a minimum of 10,000 cells were acquired and analysed using FlowJo software (Biosciences, Franklin Lakes, NJ, USA, version 10.6.2).

### 4.4. Analysis of Hypoxia in 2D and 3D Cell Cultures

2D and 3D cell cultures were treated with 1μM pimonidazole (Hypoxyprobe™ Kit Hypoxyprobe, Burlington, MA, USA) for 72 h prior to harvesting at day 7 and 14, respectively, in 70% ethanol. Samples were dissociated into a single cell suspension and incubated in 200 μL of 0.05% Triton-PBS containing a 1:200 dilution of anti-PIMO-APC or control antibody for 45 min at 4 °C. The samples were washed twice in cold PBS and followed by flow cytometric analysis (BD C6 Accuri). Data were analysed using FlowJo software (Biosciences, version 10.6.2).

### 4.5. Analysis of Reactive Oxygen Species

The levels of reactive oxygen species (ROS) were detected using 2′,7′-dichlorodihydrofluorescein diacetate (DCFH-DA, D399) (ThermoFisher Scientific, Cramlington, UK). Two-dimensional and three-dimensional cells were cultured and harvested on day 7 and 14, respectively. The cell pellets were washed and resuspended in a combination of DCFH-DA/7AAD in PBS. Samples were incubated for 15 min, followed by flow cytometric analysis (BD Accuri C6). ROS levels were quantified in live cells by gating out the 7AAD-negative cell population. Data were analysed using FlowJo software (Biosciences, version 10.6.2).

### 4.6. Analysis of Stem Cell Markers

Two-dimensional and three-dimensional cells were cultured and harvested at day 7 and 14, respectively. The cell pellets were resuspended and transferred equally into four eppendorf tubes and then mock or control, CD133-PE (Miltenyi Biotec, Bergisch Gladbach, Germany, 130-113-108) and/or CD44-APC (Miltenyi Biotec, 130-113-338) antibody treated for 20 min at 4 °C. Samples were filtered using a 70 µm filter (FlowmiTM) and 10,000 live cells were analysed using a flow cytometer (BD C6 Accuri) and using FlowJo software (Biosciences, version 10.6.2).

### 4.7. Generation and Preparation of 2D and 3D Samples for Metabolomics Analysis

G7 and R24 GBM cell lines were seeded in T75 flasks under identical cell culture media to generate 2D and 3D spheroid cultures, which were harvested on day 7 and day 14, respectively. Accurate wet cell pellet mass was determined, and samples were stored at −80 °C. For robustness, up to 8 biological replicate samples were generated under each condition.

All samples were processed in a single batch on ice and homogenised in 1000 μL of cold extraction solution consisting of methanol, acetonitrile, and water (ratio 5:3:2, repsectively). Each sample was sonicated 5 times for 30 ss (Soniprep 1500 MSE, Heathfield, UK) and then centrifuged at 18,000× *g* for 20 min at 4 °C. Negative quality control (QC) samples contained extraction solution. The supernatants were transferred into new eppendorf tubes, evaporated using a Speedvac (Labconco, Kansas City, MO, USA) at 23 °C overnight, and stored at −80 °C. Prior to LC-MS analysis, samples were resuspended in 100 μL methanol water (3:1). Samples were vortexed for 10 s, sonicated and then centrifugated for 20 min at 20,000× *g* at 4 °C. A total of 85 μL of supernatent was transferred into mass chromatography vials with 200 μL inserts and sealed with PTFE/silicone caps. QC samples were prepared by pooling 10 μL of 2D or 3D samples for analytical stability assessments.

### 4.8. High Performance Liquid Chromatography (HPLC) Mass Spectrometry (HPLC-MS)-Based Metabolomics Analysis

Metabolite characterisations were performed on a Thermo QExactive quadrupole-orbitrap mass spectrometer (Aligent technologies Inc., Santa Clara, CA, USA) equipped with a Dionex nano-flow HPLC system with an auto-sampler. Samples were separated on the hydrophilic interaction liquid chromatography (HILIC) column (1.8 μm, 2.1 × 100 mm) using a mobile phase consisting of Solvent A: Acetonitrile with 0.1% formic acid (*v*/*v*) and solvent B: 0.1% formic acid at a flow rate of 0.4 mL/min. The injection volume was set to 10 μL, and the autosampler was maintained at 4°C. All samples were subject to electrospray ionisation in both positive and negative ion modes, with a spray voltage of 3.8 and 3.5 kV, respectively. High purity nitrogen was used as a nebulising gas. Samples on the autosampler were injected randomly to eliminate bias. The QC sample and blank solvent were analysed multiple times during the run to ensure reproducibility.

The initial LC-MS raw data was converted to a mzXML/mzMatch profile. The data were processed with the MassHunter Qualitative Analysis/XCMS, including untargeted peak finding, annotation of related peaks and noise filtering. A data matrix comprising of the *m*/*z* value, peak area and retention time was obtained. Threshold values for mass and chromatographic peak heights were set to 1500 and 10,000 counts, respectively. For a molecular feature, two mass peaks were required, and the peak spacing tolerance for isotope peaks was 0.0025 *m*/*z*+ 7 parts per million (ppm). METLIN metabolite database was then used to identify putative metabolites by selecting ionisation mode, for multiple ion species with a tolerance of 10 ppm. 

### 4.9. Metabolomics Data Analysis

Metabolomics data analysis was performed using MetaboAnalyst^®^ 4.0 (http://www.metaboanalyst.ca/). Peak intensity values for each *m*/*z* value were uploaded in CSV format, followed by removal of features with >50% missing values. The data were scaled (normalized) before statistical analysis. Variables with relative standard deviations (RSDs) > 30% were removed. The MetaboAnalyst data analysis pipeline was used to conduct Principal Component Analysis (PCA) and Partial Least Squares Discriminant Analysis (PLS-DA), generate heatmaps, dendrograms, and perform hierarchical clustering of samples and features.

Only significantly altered metabolites were identified based on variable influence on projection (VIP) > 1.0, *p* < 0.05, and FDR ≤ 0.05. VIP values were acquired from the OPLS-DA model, and *p*-values were obtained from one-way ANOVA with Tukey’s post-hoc test. The false discovery rate (FDR) was used to adjust *p*-values during multiple hypothesis testing to reduce the likelihood of false positives. Significant metabolites were identified by comparing the *m*/*z* values with reference standards, including those in open-source libraries such as the Human Metabolome Database (HMDB) (http://www.hmdb.ca), with a molecular tolerance set at 20 ppm. 

The list of putative metabolites was uploaded to the Metabolomic Pathway Analysis (MetPA) tool, which utilizes the Kyoto Encyclopedia of Genes and Genomes (KEGG) database. The human pathway library was analysed to identify specific metabolic pathways associated with the putative metabolites. Additionally, enrichment analysis was performed to identify broader functional or biochemical categories enriched with significant changes in metabolite abundance using Metaboanalyst.

### 4.10. Statistical Analysis

Various statistical methods were used to analyze LC/MS metabolomic datasets. All samples were normalized according to their wet cell pellet mass. Principal component analysis (PCA) and partial least squares–discriminate analysis (PLS–DA) were performed using MetaboAnalyst 4.0 (https://metaboanalyst.ca/; accessed on 29 February 2019). Supervised and unsupervised statistical analyses were performed using PCA and PLS–DA, respectively, to identify differences between 2D and 3D cultures. The most weighted metabolites that separated 2D and 3D cultures were identified by variable importance in projection (VIP) score. Student’s *t* test was used to measure statistical differences in specific metabolite concentrations between 2D and 3D groups. *p* < 0.05 was considered statistically significant. Graphs and statistical analysis were performed using GraphPad Prism (version 10.3.0).

### 4.11. LC-MS Validation of Metabolites

Validation of metabolites in 2D and 3D G7 and R24 cultures were performed on a Thermo Scientific (Hemel Hempstead, UK) Vanquish Liquid chromatography Front end connected to IDX High Resolution Mass Spectrometer system. Samples were extracted as described earlier. MS data were acquired using the AcquieX acquisition workflow. MS orbitrap detector operating parameters were as follows: MS1 mass resolution 60 K, for MS2 30 K collisional stepped energy (HCD) 20, 35, 50 in step collision mode. The mass scan range: 100–1000 *m*/*z*, RF lens: 35%, AGC target mode custom, normalised AGC target was set to 25% (100% = 3 × 10^6^) with maximum injection time of 50 ms. The Intensity threshold was set to 2 × 10^4^. All data were acquired in profile mode. A corresponding extraction blank was used to create a background exclusion list, and a pooled QC were used to create the inclusion list.

Hydrophilic Liquid Interaction Chromatography (HILIC) and chromatographic separation were performed using a Waters Acquity UPLC BEH amide column (2.1 × 150 mm with particle size of 1.7 μm) and operating at 65 °C with a flow rate of 200 μL/min. The LC gradient consists of a binary buffer system, buffer A: 95%/5% (LC/MS grade water/ACN) and Buffer B: 90/10% (ACN/water), both containing 10 mM ammonium formate additives. Independent buffer systems were used for positive and negative mode, respectively. For positive mode, the pH of buffers were adjusted using 0.1% formic acid, and for negative mode, 0.1% Ammonia solution. The LC gradient was the same for both polarities, 95% B at T0 was held for 2 min, then linearly decreased to 50% B at 11 min, held for 4.5 min, and returned to starting conditions and held for 4.5 min further (column stabilization). The total run was approximately 21.5 min, injection to injection. Injection volume used: positive mode 2 μL and negative mode 3 μL. The voltages applied for positive mode and negative mode were 3.5 kV and 2.5 kV, respectively. The HESI conditions for 200 μL/min were as follows: Sheath Gas: 35, Aux Gas 7 and Sweep Gas of 0. Ion Transfer tube Temp: 300 °C and Vaporizer Temp 275 °C. The voltage applied for positive mode and negative mode was 3.5 kV and 2.5 kV respectively. 

The HILIC positive and negative data sets were processed via Compound Discoverer 3.2 with database to online *m*/*z* cloud database: mass tolerance 10 ppm, maximum shift 0.3 min, alignment model adaptive curve, minimum intensity 500 K, S/N threshold 3, compound consolidation, mass tolerance 10 ppm, RT tolerance 0.3 min. Database matching was performed at MS2 level with a similar index of ≥70%.

### 4.12. Effect of Hydralazine on 2D and 3D Cell Proliferation

G7 and R24 GBM cell lines were seeded at 5000 cells/well in a matrigel-coated 96-well plate. Following 24 h incubation, the wells were subject to control (sterile water) or hydralazine treatment for 168 h. Live cell images were acquired using the CELLCYTE X imaging platform (CYTENA), and 2D cell proliferation was determined by calculating well confluency percentage. Three-dimensional spheroid cultures were generated as described earlier and subjected to control or hydralazine treatments. Images of spheroid cultures were taken on day 3, 5, and 7, and spheroid volume was analysed using ImageJ.

### 4.13. Neurosphere Formation Assay

G7 and R24 cells were seeded at 10 cells/well in a 96 well plate and subject to control or hydralazine treatment. Plates were incubated at 37 °C for 28 days followed by a manual count of the number of neurosphere per well using an optical microscope at 4× magnification. The percentage of neurosphere formation in the drug treated wells was calculated relative to the control treated wells.

### 4.14. Western Blot Analysis

Western blotting was performed as described previously [5]. Briefly, 2D and 3D cell cultures were harvested from T75 flasks and subject to protein extraction using a sodium dodecyl sulphate (SDS) lysis buffer containing protease and phosphatase cocktail inhibitors (PPCI) (Sigma-Aldrich), and sonicated. Pierce™ BCA protein assay kit (Thermo Scientific) was used to determine protein concentrations, and 50 μg of protein sample was separated on SDS-polyacrylamide gel electrophoresis (SDS-PAGE) and transferred to nitrocellulose membranes. The membranes were blocked and incubated with primary antibodies Nestin (Abcam, 6320), SOX2 (Abcam, 75485), and GAPDH (Santa Cruz, Dallas, TX, USA, 47724) overnight at 4 °C and probed with secondary HRP conjugated anti-mouse IgG (Cell signaling, Leiden, Netherlands, 7076S) for 1.5 h at room temperature. Proteins were visualised using enhanced chemiluminescence (Bio-Rad Clarity Western ECL substrate) and captured using a Bio-Rad imaging platform (Bio-Rad ChemiDoc Imaging System, Hercules, CA, USA). Protein bands were quantified with ImageJ software (https://imagej.net/ij/) and normalised to GAPDH levels.

## 5. Conclusions

To the best of our knowledge, this is the first publication that links the vitamin B6 pathway to glioblastoma cell survival. Crucially, using a 3D spheroid model that recapitulates key hallmarks of in vivo tumours, we have shown that targeting the vitamin B6 pathway using hydralazine leads to GBM cell death. Future studies should consider the development of small molecule inhibitors that target key strategic points in the vitamin B6 pathway for therapeutic exploitation in GBMs.

## Data Availability

Data is contained within the article or Supplementary Material.

## Supplementary Materials

The following supporting information can be downloaded at: https://www.mdpi.com/article/10.3390/ijms251910428/s1.

## References

[1] Stupp R., Hegi M.E., Mason W.P., van den Bent M.J., Taphoorn M.J.B., Janzer R.C., Ludwin S.K., Allgeier A., Fisher B., Belanger K. (2009). Effects of Radiotherapy with Concomitant and Adjuvant Temozolomide versus Radiotherapy Alone on Survival in Glioblastoma in a Randomised Phase III Study: 5-Year Analysis of the EORTC-NCIC Trial. Lancet Oncol..

[2] Patel A.P., Tirosh I., Trombetta J.J., Shalek A.K., Gillespie S.M., Wakimoto H., Cahill D.P., Nahed B.V., Curry W.T., Martuza R.L. (2014). Single-Cell RNA-Seq Highlights Intratumoral Heterogeneity in Primary Glioblastoma. Science.

[3] Behnan J., Finocchiaro G., Hanna G. (2019). The Landscape of the Mesenchymal Signature in Brain Tumours. Brain.

[4] Bao S., Wu Q., McLendon R.E., Hao Y., Shi Q., Hjelmeland A.B., Dewhirst M.W., Bigner D.D., Rich J.N. (2006). Glioma Stem Cells Promote Radioresistance by Preferential Activation of the DNA Damage Response. Nature.

[5] Ahmed S.U., Carruthers R., Gilmour L., Yildirim S., Watts C., Chalmers A.J. (2015). Selective Inhibition of Parallel DNA Damage Response Pathways Optimizes Radiosensitization of Glioblastoma Stem-like Cells. Cancer Res..

[6] Hanahan D., Weinberg R.A. (2011). Hallmarks of Cancer: The next Generation. Cell.

[7] Dong Y., Tu R., Liu H., Qing G. (2020). Regulation of Cancer Cell Metabolism: Oncogenic MYC in the Driver’s Seat. Signal Transduct. Target. Ther..

[8] Kumar R., Mishra A., Gautam P., Feroz Z., Vijayaraghavalu S., Likos E.M., Shukla G.C., Kumar M. (2022). Metabolic Pathways, Enzymes, and Metabolites: Opportunities in Cancer Therapy. Cancers.

[9] Stine Z.E., Schug Z.T., Salvino J.M., Dang C.V. (2022). Targeting Cancer Metabolism in the Era of Precision Oncology. Nat. Rev. Drug Discov..

[10] Han S., Liu Y., Cai S.J., Qian M., Ding J., Larion M., Gilbert M.R., Yang C. (2020). IDH Mutation in Glioma: Molecular Mechanisms and Potential Therapeutic Targets. Br. J. Cancer.

[11] Murugan A.K., Alzahrani A.S. (2022). Isocitrate Dehydrogenase IDH1 and IDH2 Mutations in Human Cancer: Prognostic Implications for Gliomas. Br. J. Biomed. Sci..

[12] Reitman Z.J., Jin G., Karoly E.D., Spasojevic I., Yang J., Kinzler K.W., He Y., Bigner D.D., Vogelstein B., Yan H. (2011). Profiling the Effects of Isocitrate Dehydrogenase 1 and 2 Mutations on the Cellular Metabolome. Proc. Natl. Acad. Sci. USA.

[13] Intlekofer A.M., Dematteo R.G., Venneti S., Finley L.W.S., Lu C., Judkins A.R., Rustenburg A.S., Grinaway P.B., Chodera J.D., Cross J.R. (2015). Hypoxia Induces Production of L-2-Hydroxyglutarate. Cell Metab..

[14] Kickingereder P., Sahm F., Radbruch A., Wick W., Heiland S., von Deimling A., Bendszus M., Wiestler B. (2015). IDH Mutation Status Is Associated with a Distinct Hypoxia/Angiogenesis Transcriptome Signature Which Is Non-Invasively Predictable with rCBV Imaging in Human Glioma. Sci. Rep..

[15] Givechian K.B., Garner C., Benz S., Rabizadeh S., Soon-Shiong P. (2021). Glycolytic Expression in Lower-Grade Glioma Reveals an Epigenetic Association between IDH Mutation Status and PDL1/2 Expression. Neuro-Oncol. Adv..

[16] Pathmanapan S., Poon R., De Renshaw T.B., Nadesan P., Nakagawa M., Seesankar G.A., Loe A.K.H., Zhang H.H., Guinovart J.J., Duran J. (2023). Mutant IDH Regulates Glycogen Metabolism from Early Cartilage Development to Malignant Chondrosarcoma Formation. Cell Rep..

[17] Quartuccio N., Laudicella R., Vento A., Pignata S., Mattoli M.V., Filice R., Comis A.D., Arnone A., Baldari S., Cabria M. (2020). The Additional Value of 18F-FDG PET and MRI in Patients with Glioma: A Review of the Literature from 2015 to 2020. Diagnostics.

[18] Son B., Lee S., Kim H., Kang H., Jeon J., Jo S., Seong K.M., Lee S.-J., Youn H., Youn B. (2020). Decreased FBP1 Expression Rewires Metabolic Processes Affecting Aggressiveness of Glioblastoma. Oncogene.

[19] Sohrabi A., Lefebvre A.E.Y.T., Harrison M.J., Condro M.C., Sanazzaro T.M., Safarians G., Solomon I., Bastola S., Kordbacheh S., Toh N. (2023). Microenvironmental Stiffness Induces Metabolic Reprogramming in Glioblastoma. Cell Rep..

[20] Patil M.D., Bhaumik J., Babykutty S., Banerjee U.C., Fukumura D. (2016). Arginine Dependence of Tumor Cells: Targeting a Chink in Cancer’s Armor. Oncogene.

[21] Sawicka M.M., Sawicki K., Łysoń T., Polityńska B., Miltyk W. (2022). Proline Metabolism in Malignant Gliomas: A Systematic Literature Review. Cancers.

[22] Chen J., Cui L., Lu S., Xu S. (2024). Amino Acid Metabolism in Tumor Biology and Therapy. Cell Death Dis..

[23] Mörén L., Bergenheim A.T., Ghasimi S., Brännström T., Johansson M., Antti H. (2015). Metabolomic Screening of Tumor Tissue and Serum in Glioma Patients Reveals Diagnostic and Prognostic Information. Metabolites.

[24] Tardito S., Oudin A., Ahmed S.U., Fack F., Keunen O., Zheng L., Miletic H., Sakariassen P.Ø., Weinstock A., Wagner A. (2015). Glutamine Synthetase Activity Fuels Nucleotide Biosynthesis and Supports Growth of Glutamine-Restricted Glioblastoma. Nat. Cell Biol..

[25] Huang J., Weinstein S.J., Kitahara C.M., Karoly E.D., Sampson J.N., Albanes D. (2017). A Prospective Study of Serum Metabolites and Glioma Risk. Oncotarget.

[26] Mörén L., Perryman R., Crook T., Langer J.K., Oneill K., Syed N., Antti H. (2018). Metabolomic Profiling Identifies Distinct Phenotypes for ASS1 Positive and Negative GBM. BMC Cancer.

[27] Masters J.R. (2000). Human Cancer Cell Lines: Fact and Fantasy. Nat. Rev. Mol. Cell Biol..

[28] Mirabelli P., Coppola L., Salvatore M. (2019). Cancer Cell Lines Are Useful Model Systems for Medical Research. Cancers.

[29] Jensen C., Teng Y. (2020). Is It Time to Start Transitioning From 2D to 3D Cell Culture?. Front. Mol. Biosci..

[30] Witusik-Perkowska M., Rieske P., Hułas-Bigoszewska K., Zakrzewska M., Stawski R., Kulczycka-Wojdala D., Bieńkowski M., Stoczyńska-Fidelus E., Grešner S.M., Piaskowski S. (2011). Glioblastoma-Derived Spheroid Cultures as an Experimental Model for Analysis of EGFR Anomalies. J. Neurooncol..

[31] Akay M., Hite J., Avci N.G., Fan Y., Akay Y., Lu G., Zhu J.-J. (2018). Drug Screening of Human GBM Spheroids in Brain Cancer Chip. Sci. Rep..

[32] Paolillo M., Comincini S., Schinelli S. (2021). In Vitro Glioblastoma Models: A Journey into the Third Dimension. Cancers.

[33] Rybin M.J., Ivan M.E., Ayad N.G., Zeier Z. (2021). Organoid Models of Glioblastoma and Their Role in Drug Discovery. Front. Cell. Neurosci..

[34] Manikandan C., Jaiswal A.K. (2023). Scaffold-Based Spheroid Models of Glioblastoma Multiforme and Its Use in Drug Screening. Biotechnol. Bioeng..

[35] Riffle S., Pandey R.N., Albert M., Hegde R.S. (2017). Linking Hypoxia, DNA Damage and Proliferation in Multicellular Tumor Spheroids. BMC Cancer.

[36] Raskin N.H., Fishman R.A. (1965). Pyridoxine-Deficiency Neuropathy Due to Hydralazine. N. Engl. J. Med..

[37] Vidrio H. (1990). Interaction with Pyridoxal as a Possible Mechanism of Hydralazine Hypotension. J. Cardiovasc. Pharmacol..

[38] Jung J., Zhang Y., Celiku O., Zhang W., Song H., Williams B.J., Giles A.J., Rich J.N., Abounader R., Gilbert M.R. (2019). Mitochondrial NIX Promotes Tumor Survival in the Hypoxic Niche of Glioblastoma. Cancer Res..

[39] Krawczynski K., Godlewski J., Bronisz A. (2020). Oxidative Stress—Part of the Solution or Part of the Problem in the Hypoxic Environment of a Brain Tumor. Antioxidants.

[40] Peixoto J., Janaki-Raman S., Schlicker L., Schmitz W., Walz S., Winkelkotte A.M., Herold-Mende C., Soares P., Schulze A., Lima J. (2021). Integrated Metabolomics and Transcriptomics Analysis of Monolayer and Neurospheres from Established Glioblastoma Cell Lines. Cancers.

[41] Phang J.M. (2019). Proline Metabolism in Cell Regulation and Cancer Biology: Recent Advances and Hypotheses. Antioxid. Redox Signal..

[42] Elia I., Broekaert D., Christen S., Boon R., Radaelli E., Orth M.F., Verfaillie C., Grünewald T.G.P., Fendt S.-M. (2017). Proline Metabolism Supports Metastasis Formation and Could Be Inhibited to Selectively Target Metastasizing Cancer Cells. Nat. Commun..

[43] Liu W., Glunde K., Bhujwalla Z.M., Raman V., Sharma A., Phang J.M. (2012). Proline Oxidase Promotes Tumor Cell Survival in Hypoxic Tumor Microenvironments. Cancer Res..

[44] Chen C.-L., Hsu S.-C., Ann D.K., Yen Y., Kung H.-J. (2021). Arginine Signaling and Cancer Metabolism. Cancers.

[45] Syed N., Langer J., Janczar K., Singh P., Lo Nigro C., Lattanzio L., Coley H.M., Hatzimichael E., Bomalaski J., Szlosarek P. (2013). Epigenetic Status of Argininosuccinate Synthetase and Argininosuccinate Lyase Modulates Autophagy and Cell Death in Glioblastoma. Cell Death Dis..

[46] Wu G., Bazer F.W., Davis T.A., Kim S.W., Li P., Rhoads J.M., Satterfield M.C., Smith S.B., Spencer T.E., Yin Y. (2009). Arginine Metabolism and Nutrition in Growth, Health and Disease. Amino Acids.

[47] Geeraerts S.L., Heylen E., De Keersmaecker K., Kampen K.R. (2021). The Ins and Outs of Serine and Glycine Metabolism in Cancer. Nat. Metab..

[48] Ganini C., Amelio I., Bertolo R., Candi E., Cappello A., Cipriani C., Mauriello A., Marani C., Melino G., Montanaro M. (2021). Serine and One-Carbon Metabolisms Bring New Therapeutic Venues in Prostate Cancer. Discov. Oncol..

[49] Shunxi W., Xiaoxue Y., Guanbin S., Li Y., Junyu J., Wanqian L. (2023). Serine Metabolic Reprogramming in Tumorigenesis, Tumor Immunity, and Clinical Treatment. Adv. Nutr..

[50] Singh C., Sharma A., Hoppe G., Song W., Bolok Y., Sears J.E. (2018). 3-Hydroxypyruvate Destabilizes Hypoxia Inducible Factor and Induces Angiostasis. Investig. Ophthalmol. Vis. Sci..

[51] Labuschagne C.F., van den Broek N.J.F., Mackay G.M., Vousden K.H., Maddocks O.D.K. (2014). Serine, but Not Glycine, Supports One-Carbon Metabolism and Proliferation of Cancer Cells. Cell Rep..

[52] Mentch S.J., Locasale J.W. (2016). One Carbon Metabolism and Epigenetics: Understanding the Specificity. Ann. N. Y. Acad. Sci..

[53] Jeon H., Kim J.H., Lee E., Jang Y.J., Son J.E., Kwon J.Y., Lim T.-G., Kim S., Park J.H.Y., Kim J.-E. (2016). Methionine Deprivation Suppresses Triple-Negative Breast Cancer Metastasis in Vitro and in Vivo. Oncotarget.

[54] Zhang Q., Li W. (2022). Correlation between Amino Acid Metabolism and Self-Renewal of Cancer Stem Cells: Perspectives in Cancer Therapy. World J. Stem Cells.

[55] Palanichamy K., Thirumoorthy K., Kanji S., Gordon N., Singh R., Jacob J.R., Sebastian N., Litzenberg K.T., Patel D., Bassett E. (2016). Methionine and Kynurenine Activate Oncogenic Kinases in Glioblastoma, and Methionine Deprivation Compromises Proliferation. Clin. Cancer Res..

[56] Pascale R.M., Peitta G., Simile M.M., Feo F. (2019). Alterations of Methionine Metabolism as Potential Targets for the Prevention and Therapy of Hepatocellular Carcinoma. Medicina.

[57] Zgheib R., Battaglia-Hsu S.-F., Hergalant S., Quéré M., Alberto J.-M., Chéry C., Rouyer P., Gauchotte G., Guéant J.-L., Namour F. (2019). Folate Can Promote the Methionine-Dependent Reprogramming of Glioblastoma Cells towards Pluripotency. Cell Death Dis..

[58] Jin J., Byun J.-K., Choi Y.-K., Park K.-G. (2023). Targeting Glutamine Metabolism as a Therapeutic Strategy for Cancer. Exp. Mol. Med..

[59] Martins F., Gonçalves L.G., Pojo M., Serpa J. (2020). Take Advantage of Glutamine Anaplerosis, the Kernel of the Metabolic Rewiring in Malignant Gliomas. Biomolecules.

[60] Jin L., Alesi G., Kang S. (2016). Glutaminolysis as a Target for Cancer Therapy. Oncogene.

[61] Mukherjee P., Augur Z.M., Li M., Hill C., Greenwood B., Domin M.A., Kondakci G., Narain N.R., Kiebish M.A., Bronson R.T. (2019). Therapeutic Benefit of Combining Calorie-Restricted Ketogenic Diet and Glutamine Targeting in Late-Stage Experimental Glioblastoma. Commun. Biol..

[62] Koch K., Hartmann R., Tsiampali J., Uhlmann C., Nickel A.-C., He X., Kamp M.A., Sabel M., Barker R.A., Steiger H.-J. (2020). A Comparative Pharmaco-Metabolomic Study of Glutaminase Inhibitors in Glioma Stem-like Cells Confirms Biological Effectiveness but Reveals Differences in Target-Specificity. Cell Death Discov..

[63] De Los Santos-Jiménez J., Rosales T., Ko B., Campos-Sandoval J.A., Alonso F.J., Márquez J., DeBerardinis R.J., Matés J.M. (2023). Metabolic Adjustments Following Glutaminase Inhibition by CB-839 in Glioblastoma Cell Lines. Cancers.

[64] Rabinovich S., Adler L., Yizhak K., Sarver A., Silberman A., Agron S., Stettner N., Sun Q., Brandis A., Helbling D. (2015). Diversion of Aspartate in ASS1-Deficient Tumours Fosters de Novo Pyrimidine Synthesis. Nature.

[65] Mullen N.J., Singh P.K. (2023). Nucleotide Metabolism: A Pan-Cancer Metabolic Dependency. Nat. Rev. Cancer.

[66] Jung J., Kim L.J., Wang X., Wu Q., Sanvoranart T., Hubert C.G., Prager B.C., Wallace L.C., Jin X., Mack S.C. (2017). Nicotinamide Metabolism Regulates Glioblastoma Stem Cell Maintenance. JCI Insight.

[67] Wang X., Yang K., Xie Q., Wu Q., Mack S.C., Shi Y., Kim L.J.Y., Prager B.C., Flavahan W.A., Liu X. (2017). Purine Synthesis Promotes Maintenance of Brain Tumor Initiating Cells in Glioma. Nat. Neurosci..

[68] Talib W.H., Ahmed Jum’AH D.A., Attallah Z.S., Jallad M.S., Al Kury L.T., Hadi R.W., Mahmod A.I. (2024). Role of Vitamins A, C, D, E in Cancer Prevention and Therapy: Therapeutic Potentials and Mechanisms of Action. Front. Nutr..

[69] Jabbari P., Yazdanpanah O., Benjamin D.J., Kalebasty A.R. (2024). Supplement Use and Increased Risks of Cancer: Unveiling the Other Side of the Coin. Cancers.

[70] Stach K., Stach W., Augoff K. (2021). Vitamin B6 in Health and Disease. Nutrients.

[71] Ellershaw D.C., Gurney A.M. (2001). Mechanisms of Hydralazine Induced Vasodilation in Rabbit Aorta and Pulmonary Artery. Br. J. Pharmacol..

[72] Kang H., Lee H., Kim D., Kim B., Kang J., Kim H.Y., Youn H., Youn B. (2022). Targeting Glioblastoma Stem Cells to Overcome Chemoresistance: An Overview of Current Therapeutic Strategies. Biomedicines.

[73] Chavez-Blanco A., Perez-Plasencia C., Perez-Cardenas E., Carrasco-Legleu C., Rangel-Lopez E., Segura-Pacheco B., Taja-Chayeb L., Trejo-Becerril C., Gonzalez-Fierro A., Candelaria M. (2006). Antineoplastic Effects of the DNA Methylation Inhibitor Hydralazine and the Histone Deacetylase Inhibitor Valproic Acid in Cancer Cell Lines. Cancer Cell Int..

[74] Song Y., Zhang C. (2009). Hydralazine Inhibits Human Cervical Cancer Cell Growth in Vitro in Association with APC Demethylation and Re-Expression. Cancer Chemother. Pharmacol..

[75] Knowles H.J., Tian Y.-M., Mole D.R., Harris A.L. (2004). Novel Mechanism of Action for Hydralazine. Circ. Res..

[76] Graça I., Sousa E.J., Costa-Pinheiro P., Vieira F.Q., Torres-Ferreira J., Martins M.G., Henrique R., Jerónimo C. (2014). Anti-Neoplastic Properties of Hydralazine in Prostate Cancer. Oncotarget.

[77] Lopes N., Pacheco M.B., Soares-Fernandes D., Correia M.P., Camilo V., Henrique R., Jerónimo C. (2021). Hydralazine and Enzalutamide: Synergistic Partners against Prostate Cancer. Biomedicines.

[78] Pacheco M.B., Camilo V., Lopes N., Moreira-Silva F., Correia M.P., Henrique R., Jerónimo C. (2021). Hydralazine and Panobinostat Attenuate Malignant Properties of Prostate Cancer Cell Lines. Pharmaceuticals.

[79] Chen C.-C., Li B., Millman S.E., Chen C., Li X., Morris J.P., Mayle A., Ho Y.-J., Loizou E., Liu H. (2020). Vitamin B6 Addiction in Acute Myeloid Leukemia. Cancer Cell.

