# Supplementary material for: Vitamin B6 Pathway Maintains Glioblastoma Cell Survival in 3D Spheroid Cultures
Source: Int J Mol Sci. 2024 Sep 27;25(19):10428. doi: 10.3390/ijms251910428 (PMC11476381; doi:10.3390/ijms251910428)
Supplement: Supplementary file 1 [file ijms-25-10428-s001.zip › ijms-3211564-supplementary.pdf]

Supplementary Figure S1

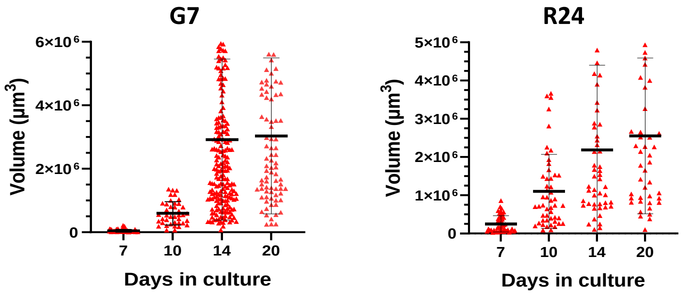

Volume of individual spheroids in G7 and R24 3D cultures on day 7, 10, 14 and 20. Mean spheroid volume is shown by horizontal line. Volume of 30-40 spheroids from a single experiment is shown.

Supplementary Table S1

Table shows 250 significantly altered features identified in both G7 and R24 GBM cell lines consisting of 154-up (green) and 96-downregulated (grey) in 3D spheroids compared to 2D cultures. M/Z value and the associated VIP, p-value, FDR and average of fold change (FC) and log_2FC for each feature is shown.

m/z value	VIP	p.value	FDR	log_2(FC)	Fold Change
374.1579	1.48	7.15E-08	0.0000014	1.55	35.74
234.1085	1.47	1.19E-05	0.0001022	1.44	27.66
524.2794	1.38	0.000138	0.0007925	1.39	24.48
584.2651	1.33	2.51E-15	0.0000000	1.37	23.22
631.2608	1.46	1.49E-10	0.0000000	1.30	20.03
454.182	1.45	1.19E-06	0.0000081	1.30	19.97
248.124	1.66	9.2E-09	0.0000002	1.23	16.82
1354.661	1.40	7.72E-05	0.0004787	1.18	15.21
655.3478	1.37	1.14E-12	0.0000000	1.14	13.95
265.132	1.43	6.5E-11	0.0000000	1.14	13.69
771.3103	1.45	1.3E-09	0.0000000	1.11	12.95
226.1073	1.63	1.39E-14	0.0000000	1.08	11.94
100.0279	1.26	1.21E-16	0.0000000	1.07	11.78
476.1643	1.55	1.33E-07	0.0000023	1.07	11.75
197.0208	1.68	2.88E-05	0.0002089	1.05	11.31
546.2616	1.23	3.24E-05	0.0002307	1.04	10.94
497.0148	1.19	1.02E-08	0.0000003	1.03	10.65
1092.523	1.69	8.55E-08	0.0000005	1.03	10.64
645.2764	1.55	1.24E-05	0.0001053	1.01	10.25
245.113	1.73	1.68E-11	0.0000000	0.97	9.42
808.4002	1.88	0.005889	0.0176055	0.97	9.24
275.1127	1.78	1.09E-11	0.0000000	0.96	9.07
519.2433	1.39	3.17E-06	0.0000336	0.92	8.35
314.1362	1.36	9.25E-09	0.0000002	0.92	8.34
703.5168	1.44	3.74E-06	0.0000382	0.92	8.29
263.1238	1.52	6.05E-08	0.0000012	0.91	8.20
117.0543	1.41	6.37E-12	0.0000000	0.91	8.15
361.1262	1.31	5.81E-05	0.0003747	0.91	8.10
305.1232	1.60	1.58E-05	0.0001277	0.89	7.75
274.0685	1.25	6.55E-06	0.0000611	0.88	7.65
431.1652	1.68	6.3E-06	0.0000598	0.88	7.56
265.0472	1.71	3.28E-07	0.0000030	0.88	7.52
427.1197	1.41	8.8E-06	0.0000792	0.87	7.37
308.1193	1.36	4.59E-10	0.0000000	0.86	7.22
809.2664	1.50	5.6E-09	0.0000002	0.86	7.21
237.1006	1.49	6.25E-07	0.0000082	0.84	6.90
131.07	1.48	5.65E-06	0.0000542	0.84	6.89
511.9185	1.58	3.67E-11	0.0000000	0.83	6.74

322.2112	1.28	5.15E-09	0.0000001	0.83	6.74
580.9762	1.43	2.05E-05	0.0001449	0.82	6.57
481.0408	1.58	1.23E-10	0.0000000	0.80	6.36
565.0025	1.30	3.57E-05	0.0002479	0.79	6.22
380.0874	1.74	6.85E-07	0.0000090	0.79	6.16
490.1983	1.39	0.003675	0.0121103	0.79	6.14
175.0041	1.29	9.38E-05	0.0005631	0.77	5.94
1130.004	1.40	1.66E-05	0.0001313	0.76	5.81
204.955	1.23	0.000788	0.0033345	0.76	5.78
443.0843	1.35	3.5E-08	0.0000007	0.76	5.75
232.1068	1.23	0.001803	0.0068095	0.76	5.69
146.0696	1.67	0.005094	0.0157055	0.74	5.49
343.9954	1.42	0.001417	0.0055725	0.74	5.47
301.2261	1.30	5.71E-08	0.0000011	0.73	5.40
248.1003	1.44	0.000388	0.0018610	0.72	5.21
407.2359	1.68	4.73E-09	0.0000001	0.71	5.16
290.0425	1.08	0.000306	0.0015356	0.71	5.14
645.169	1.63	3.82E-16	0.0000000	0.70	5.04
173.007	1.45	0.005948	0.0177000	0.70	5.02
256.0467	1.67	5.72E-09	0.0000002	0.70	4.96
306.0392	1.20	7.2E-06	0.0000663	0.69	4.91
393.209	1.52	0.000784	0.0033319	0.69	4.91
493.9668	1.32	7.4E-06	0.0000682	0.69	4.85
137.0482	1.52	2.51E-07	0.0000039	0.68	4.75
495.394	1.27	8.95E-07	0.0000113	0.67	4.71
368.1597	1.55	1.98E-12	0.0000000	0.67	4.64
247.1063	1.67	7.88E-05	0.0004872	0.67	4.63
183.0415	1.43	4.66E-05	0.0003096	0.66	4.60
294.2163	1.60	0.002294	0.0055660	0.66	4.54
549.0286	1.23	3.3E-08	0.0000007	0.65	4.51
350.1786	1.23	1.15E-06	0.0000141	0.65	4.47
318.1336	1.47	0.000347	0.0016999	0.65	4.45
526.822	1.37	1.37E-06	0.0000062	0.64	4.38
465.0665	1.67	0.004175	0.0134515	0.64	4.35
121.0257	1.87	1.03E-10	0.0000000	0.64	4.35
368.1596	1.32	3.59E-05	0.0002478	0.64	4.34
442.8602	1.62	3.8E-07	0.0000054	0.63	4.28
388.1732	1.68	1.39E-08	0.0000003	0.63	4.26
495.9443	1.55	1.18E-06	0.0000143	0.62	4.19
406.2323	1.29	0.004606	0.0146276	0.62	4.13
257.1018	1.13	0.002197	0.0054163	0.61	4.10
334.1578	1.59	2.21E-06	0.0000243	0.61	4.08
546.1939	1.52	7.05E-12	0.0000000	0.61	4.04
411.9827	1.31	3.43E-06	0.0000355	0.60	3.97
111.0439	1.53	1.25E-07	0.0000007	0.60	3.95

358.8989	1.77	0.000448	0.0020889	0.59	3.91
271.179	1.66	1.94E-06	0.0000204	0.58	3.81
315.2414	1.48	0.0013	0.0051930	0.58	3.78
273.1584	1.38	0.000277	0.0014151	0.57	3.75
119.074	1.40	0.000645	0.0028292	0.57	3.73
151.015	1.63	0.00216	0.0060064	0.56	3.66
261.1584	1.67	0.001757	0.0066650	0.56	3.66
227.0911	1.47	3.49E-07	0.0000051	0.56	3.60
273.1948	1.22	0.00286	0.0077006	0.56	3.60
467.2216	1.43	0.000638	0.0028041	0.55	3.54
298.0996	1.45	2.26E-08	0.0000005	0.54	3.48
415.1913	1.46	0.000274	0.0014018	0.54	3.46
129.043	1.34	3.22E-05	0.0002300	0.54	3.43
225.0621	1.14	7.79E-11	0.0000000	0.53	3.39
113.0594	1.25	2.8E-08	0.0000006	0.52	3.30
920.7129	1.31	2.6E-05	0.0001908	0.52	3.29
890.6486	1.39	1.11E-08	0.0000003	0.52	3.29
125.0594	1.41	0.00031	0.0015529	0.51	3.27
676.9922	1.24	0.000347	0.0010148	0.51	3.24
299.2105	1.28	3.76E-07	0.0000053	0.51	3.20
1328.494	1.29	0.000443	0.0020711	0.51	3.20
410.0059	1.59	7.51E-05	0.0004692	0.51	3.20
380.0874	1.52	0.000293	0.0014838	0.50	3.19
287.2105	1.24	0.000378	0.0018189	0.50	3.17
211.0671	1.61	0.003206	0.0109343	0.49	3.12
257.073	1.83	0.008083	0.0228016	0.49	3.09
279.0839	1.41	1.79E-05	0.0000693	0.49	3.07
460.3563	1.15	2.51E-07	0.0000039	0.48	3.04
209.0436	1.61	0.003517	0.0118219	0.48	3.02
426.3508	1.41	5.05E-07	0.0000025	0.47	2.94
329.2571	1.20	0.000563	0.0025300	0.46	2.91
304.079	1.15	6.99E-06	0.0000634	0.46	2.91
249.0732	1.19	1.02E-05	0.0000891	0.46	2.90
385.1626	1.59	3.66E-06	0.0000378	0.46	2.87
265.0472	1.74	2.73E-05	0.0000980	0.45	2.84
733.5631	1.41	0.0002	0.0010866	0.45	2.80
478.3672	1.54	0.003205	0.0074913	0.44	2.78
282.0971	0.68	0.000152	0.0008621	0.44	2.77
136.0389	0.79	6.42E-08	0.0000012	0.44	2.77
145.0855	1.49	1.79E-07	0.0000029	0.44	2.74
455.1194	1.13	0.000732	0.0031519	0.44	2.74
477.3828	1.72	1.05E-06	0.0000057	0.44	2.73
259.179	1.42	1.8E-05	0.0001413	0.43	2.72
238.0626	1.16	4.83E-06	0.0000471	0.43	2.67
759.5801	1.44	0.000391	0.0018689	0.43	2.66

1619.091	1.69	0.000214	0.0008244	0.42	2.65
285.1948	1.64	0.000754	0.0032259	0.42	2.64
782.5656	1.25	2.08E-05	0.0000840	0.42	2.63
195.0571	1.84	2.23E-05	0.0001677	0.42	2.63
425.3515	1.16	3E-05	0.0001059	0.42	2.61
261.122	1.24	0.000498	0.0022981	0.42	2.61
249.0731	1.06	3.08E-09	0.0000000	0.41	2.58
132.0904	1.14	2.7E-06	0.0000289	0.41	2.56
261.097	1.58	3.7E-09	0.0000001	0.41	2.56
181.9988	1.30	4.02E-09	0.0000001	0.40	2.54
199.0617	1.78	4.1E-06	0.0000414	0.40	2.52
325.0338	1.55	1.1E-06	0.0000051	0.40	2.52
491.3025	1.65	0.00448	0.0142842	0.40	2.50
243.0703	1.27	0.000352	0.0017190	0.38	2.42
241.0722	1.75	1.9E-06	0.0000085	0.37	2.34
545.9896	1.38	1.44E-10	0.0000000	0.37	2.33
975.1021	1.55	2.6E-06	0.0000117	0.36	2.30
193.0744	1.55	1.6E-05	0.0000596	0.35	2.26
781.5619	1.40	0.000899	0.0037291	0.35	2.23
444.3615	1.58	2.73E-09	0.0000001	0.34	2.18
310.1786	1.60	2.66E-06	0.0000287	0.34	2.17
221.073	1.76	3.78E-09	0.0000000	0.33	2.12
585.5113	1.20	0.000522	0.0023768	0.31	2.06
788.6136	1.13	0.000263	0.0009188	0.10	1.26
791.5661	1.56	0.001843	0.0069515	0.00	1.01
508.1544	1.41	1.42E-06	0.0000166	0.00	1.00
632.6316	1.57	0.000187	0.0010234	-0.01	0.97
813.6254	1.15	0.001605	0.0062159	-0.01	0.97
785.5233	1.27	2.62E-08	0.0000006	-0.02	0.96
465.1063	1.61	0.009324	0.0255345	-0.02	0.95
177.0019	1.62	0.001161	0.0047105	-0.04	0.92
691.5145	1.58	0.002657	0.0094864	-0.05	0.89
299.2833	1.59	7.89E-05	0.0004858	-0.06	0.87
222.068	1.34	8.5E-07	0.0000040	-0.09	0.81
787.6056	1.34	3.12E-06	0.0000133	-0.14	0.73
133.0201	1.78	1.67E-08	0.0000004	-0.14	0.72
486.1724	1.29	2.75E-11	0.0000000	-0.15	0.70
235.9913	1.50	0.005082	0.0137065	-0.31	0.49
694.1277	1.45	2.15E-05	0.0001595	-0.32	0.48
555.8539	1.20	4.1E-05	0.0002765	-0.34	0.45
815.639	1.58	0.000315	0.0015734	-0.35	0.44
165.0465	1.51	0.003246	0.0110160	-0.36	0.44
288.9167	1.68	7.27E-06	0.0000645	-0.37	0.42
610.0968	1.25	0.000166	0.0008670	-0.39	0.41
306.0374	1.39	0.000186	0.0010204	-0.39	0.41

368.2437	1.48	0.01276	0.0333075	-0.42	0.38
331.0888	1.52	8.96E-05	0.0005427	-0.43	0.37
557.3596	1.33	3.29E-07	0.0000030	-0.43	0.37
421.0297	1.34	0.000309	0.0013536	-0.44	0.37
499.284	1.20	2.4E-05	0.0001692	-0.45	0.36
571.1691	1.56	1.25E-05	0.0001054	-0.45	0.36
307.2879	1.53	0.001814	0.0058677	-0.45	0.36
319.249	1.40	0.000281	0.0014319	-0.45	0.35
264.0141	1.09	5.21E-05	0.0003325	-0.46	0.34
754.2355	1.20	7.7E-06	0.0000303	-0.47	0.34
481.3553	1.29	6.2E-06	0.0000589	-0.47	0.34
202.1073	1.78	7.27E-06	0.0000507	-0.48	0.33
600.3286	1.46	6.47E-05	0.0004128	-0.50	0.32
342.2279	1.39	0.001445	0.0056676	-0.50	0.32
623.8409	1.49	0.003412	0.0114482	-0.51	0.31
675.4853	1.40	9.55E-07	0.0000120	-0.51	0.31
154.0359	1.42	1.3E-08	0.0000002	-0.51	0.31
447.1368	1.41	4.83E-06	0.0000362	-0.51	0.31
457.0745	1.51	3.78E-05	0.0002591	-0.51	0.31
716.5394	1.47	0.001912	0.0071688	-0.52	0.30
321.265	1.34	0.000579	0.0025855	-0.53	0.29
148.986	1.44	0.000577	0.0025859	-0.54	0.29
234.9867	1.45	0.005033	0.0156469	-0.54	0.29
175.0487	0.83	0.000459	0.0020829	-0.54	0.29
400.3353	0.64	2.32E-05	0.0001730	-0.55	0.28
262.0783	1.25	4.04E-05	0.0001571	-0.55	0.28
956.3161	1.23	2.37E-08	0.0000002	-0.56	0.27
348.1115	1.49	3.62E-05	0.0001797	-0.56	0.27
187.0285	1.37	0.000485	0.0014448	-0.57	0.27
201.9972	1.36	0.000122	0.0004417	-0.57	0.27
281.2726	1.51	1.8E-05	0.0001414	-0.59	0.26
85.08989	1.05	1.64E-06	0.0000089	-0.59	0.26
334.0999	1.40	3.01E-07	0.0000046	-0.59	0.26
303.2545	1.46	1.29E-09	0.0000000	-0.59	0.26
332.0016	1.66	0.002937	0.0094664	-0.60	0.25
515.2953	1.65	0.001654	0.0063782	-0.61	0.24
149.0069	1.24	0.000603	0.0026794	-0.61	0.24
274.0526	1.34	1.12E-13	0.0000000	-0.63	0.24
176.0179	1.62	0.000265	0.0013619	-0.63	0.24
294.0926	1.60	0.000557	0.0024070	-0.65	0.23
178.9966	1.22	0.011705	0.0235820	-0.65	0.22
397.0939	1.52	0.000356	0.0017316	-0.65	0.22
170.0098	1.43	4.46E-05	0.0001549	-0.66	0.22
221.0907	1.37	0.003365	0.0113405	-0.67	0.21
352.1858	1.41	0.000616	0.0017038	-0.68	0.21

677.2287	1.49	0.000141	0.0005806	-0.68	0.21
159.0357	1.19	6E-14	0.0000000	-0.69	0.20
509.3862	1.44	3.15E-11	0.0000000	-0.72	0.19
183.0514	1.71	3.76E-07	0.0000019	-0.73	0.19
268.9872	1.40	0.000497	0.0022969	-0.74	0.18
375.0441	1.39	3.52E-05	0.0002402	-0.77	0.17
604.1767	1.44	0.000574	0.0024297	-0.77	0.17
141.0406	1.45	0.000644	0.0023691	-0.78	0.17
413.3515	1.53	0.000024	0.0001332	-0.78	0.16
432.1371	1.48	0.002016	0.0075029	-0.79	0.16
561.1667	1.58	1.09E-08	0.0000003	-0.81	0.16
163.0225	1.50	0.001318	0.0052289	-0.81	0.15
320.0371	1.59	2.63E-06	0.0000225	-0.81	0.15
360.1769	1.57	0.00083	0.0034870	-0.82	0.15
172.023	1.52	0.001105	0.0044959	-0.83	0.15
351.1259	1.37	7.21E-11	0.0000000	-0.83	0.15
236.0803	1.50	1.31E-13	0.0000000	-0.85	0.14
312.0292	1.10	0.000801	0.0033789	-0.86	0.14
326.0733	1.43	5.1E-10	0.0000000	-0.86	0.14
355.0718	0.52	4.37E-07	0.0000061	-0.89	0.13
435.1328	0.80	0.010139	0.0198316	-0.89	0.13
244.0418	0.99	3.68E-06	0.0000379	-0.89	0.13
429.1263	0.57	0.005968	0.0173085	-0.93	0.12
118.9965	1.11	0.001253	0.0050201	-0.95	0.11
436.0999	1.26	0.006861	0.0201353	-0.99	0.10
347.0732	0.95	1.31E-05	0.0001015	-1.03	0.09
586.1442	0.80	2.02E-05	0.0001544	-1.08	0.08
1172.288	0.64	0.002292	0.0083587	-1.10	0.08
420.1257	0.80	0.003936	0.0128155	-1.18	0.07
304.0631	1.63	4.05E-11	0.0000000	-1.30	0.05
445.1034	1.30	0.001882	0.0047565	-1.37	0.04
292.0916	1.62	1.54E-06	0.0000114	-1.81	0.02

Supplementary Table S2

Table shows all the putative metabolites identified from the 154 significantly upregulated features in 3D cultures. The maximum mass tolerance for metabolite identification (ID) was set to 20ppm. Some features led to multiple metabolite ID, whilst others did not yield an ID (blank).

m/z value	Metabolites	PPM
508.15441	L-Cystathionine	7
	Allocystathionine	7
	(2E)-Octenoyl-CoA	10
791.56605	PE (18:1(11Z)/P-18:1(9Z))	2
549.38091	LysoPC(20:1(11Z)/0:0)	2
	Cholesterol sulfate	16
165.06549	4-Pyridoxolactone	0
	Formylanthranilic acid	1
	Xanthurenic acid	2
	Picolinic acid	2
	Indole-5,6-quinone	2
	1-Aminocyclopropanecarboxylic acid	13
	Methionine sulfoxide	19
139.03613	Glutarate semialdehyde	3
	Alpha-ketoisovaleric acid	3
	L-Phosphoarginine	8
	Glucosamine 6-sulfate	18
	N-Sulfo-D-glucosamine	18
	Gentisate aldehyde	20
183.04149	Tyrosol	12
221.073		
310.1786	Methionyl-Lysine	3
444.36152	PC (24:1(15Z)/18:1(9Z))	6
975.10213	2-(a-Hydroxyethyl) thiamine diphosphate	11
146.06952	Argininosuccinic acid	6
325.03376	6-Hydroxymelatonin	4
	4-Hydroxy-2-oxoglutaric acid	20
	D-4-Hydroxy-2-oxoglutarate	20
325.13923		
132.09038		
425.35154	TG (16:1(9Z)/16:1(9Z)/18:2(9Z,12Z))	2
	PC (15:0/24:0)	11
	PE (18:0/24:0)	11
111.04389	(S)-2-Aceto-2-hydroxybutanoic acid	12
	Butyric acid	20
111.04391	(S)-2-Aceto-2-hydroxybutanoic acid	12

	Butyric acid	20
259.17903	Gamma-glutamyl-L-putrescine	10
	beta-Alanyl-L-lysine	10
136.03887	(S)-1-Pyrroline-5-carboxylate	15
	N2-Succinyl-L-ornithine	5
	5-Phosphoribosylamine	6
	1-Pyrroline-5-carboxylic acid	15
	3-Hydroxyanthranilic acid	8
	Pyruvaldehyde	15
282.09707	4-Hydroxyphenylacetylglutamine	1
478.36716		
265.04721		
385.16257	UDP-D-Xylose	5
262.13976	Glucosamine	0
249.07315	L-Aspartic acid	5
	D-Aspartic acid	5
426.35078	Glucosylceramide (d18:1/18:0)	13
	TG(18:1(9Z)/14:0/18:2(9Z,12Z))	18
209.04355	L-Fucose	19
	L-Fuculose	19
	Citraconic acid	20
	Itaconic acid	20
	2,5-Dioxopentanoate	20
279.08391	Anserine	5
	Homocarnosine	5
	Poly-g-D-glutamate	20
257.07295	CMP-N-trimethyl-2-aminoethylphosphonate	2
	L-2-Amino-3-oxobutanoic acid	6
	cis-Aconitic acid	15
	trans-Aconitic acid	15
	Dehydroascorbic acid	15
	Gluconolactone	16
	2-Keto-3-deoxy-D-gluconic acid	16
	Galactonolactone	16
	L-Gulonolactone	16
261.12204		
211.06713	L-Glutamic acid	8
	O-Acetylserine	8
	D-Glutamic acid	8
	L-4-Hydroxyglutamate semialdehyde	8
	Propinol adenylate	9
	AICAR	0
	Glutaric acid	17

111.04382	(S)-2-Aceto-2-hydroxybutanoic acid	12
	Butyric acid	20
287.21052	3-Hydroxy-N6,N6,N6-trimethyl-L-lysine	8
380.08741		
	Imidazoleacetic acid ribotide	0
	L-Dihydroorotic acid	5
	4,5-Dihydroorotic acid	16
	Lactosamine	16
146.06957	Argininosuccinic acid	7
676.99222		
125.05935	Ciliatine	1
	N2-Succinyl-L-glutamic acid 5-semialdehyde	13
920.71292	PC(22:5(7Z,10Z,13Z,16Z,19Z)/24:0)	3
113.05944	Ketoleucine	8
	(R) 2,3-Dihydroxy-3-methylvalerate	13
227.09109	(R)-Pantoate	16
177.00194		
274.12861	Pantothenol	9
415.19128	4-hydroxy-D4-neuroprostane	8
	N6-Acetyl-L-lysine	10
	N-Acetyl-L-phenylalanine	12
298.09964	CMP-N-trimethyl-2-aminoethylphosphonate	1
	L-2-Amino-3-oxobutanoic acid	4
	L-Aspartate-semialdehyde	4
	5'-Methylthioadenosine	9
275.11265	S)-2-Aceto-2-hydroxybutanoic acid	0
	L-2,4-diaminobutyric acid	4
	Iodotyrosine	10
	Hypoxanthine	14
129.04306	cis-Aconitic acid	8
	trans-Aconitic acid	8
546.1939	D-Glucosaminide	11
	Levan	16
	Dextrin	16
	D-Gal alpha 1->6D-Gal alpha 1->6D-Glucose	16
	Gentianose	16
	5-Hydroxy-N-formylkynurenine	20
	Deoxyinosine	20
334.15776	Deoxyadenosine	11
	2-Phenylacetamide	15
257.10181	Indole	12
	Guanidoacetic acid	19
388.17322	N-Acetyl-L-glutamate 5-semialdehyde	5
	Cis-zeatin-7-N-glucoside	20
	Thiamine	20

135.04126	Propane-1,3-diol	15
121.02572		
197.0208	Deoxyribose 5-phosphate	4
	Deoxyribose 1-phosphate	4
	Uridine diphosphate glucose	6
	Uridine diphosphategalactose	6
	p-Hydroxyphenylacetic acid	12
350.17859		
294.21629	LysoSM(d18:1)	15
146.0695	repeat	
247.10626	2-Hydroxycinnamic acid	6
	Phenylpyruvic acid	6
	m-Coumaric acid	6
	Enol-phenylpyruvate	6
	5-Methoxyindoleacetate	6
	Acetyl-N-formyl-5-methoxykynurenamine	8
	Alpha-N-Phenylacetyl-L-glutamine	8
	L-Asparagine	10
	Ureidopropionic acid	10
	Thiamine	16
	Pyridoxamine	20
495.39403		
137.04816	Hypoxanthine	17
129.04301	cis-Aconitic acid	8
	trans-Aconitic acid	8
	Dehydroascorbic acid	8
393.20897	11,12,15-THETA	13
256.04671	2,3-Diketo-L-gulonate	15
	Citric acid	15
	Isocitric acid	15
	Diketogulonic acid	15
	Citicoline	20
117.05437	Glutarate semialdehyde	2
	Alpha-ketoisovaleric acid	2
	(R)-2,3-Dihydroxy-isovalerate	7
	Deoxyribose	7
	D-Xylitol	12
	L-Arabitol	12
	D-Arabitol	12
645.16895		
301.22612		
308.11939		
232.10675	L-Methionine	20
322.2112	LysoPC(18:2(9Z,12Z))	7
131.06996	Lactaldehyde	2

	Methyl acetate	3
	Propionic acid	3
	Hydroxyacetone	3
	(R) 2,3-Dihydroxy-3-methylvalerate	7
	2-Aminobenzoic acid	7
427.1197	Phosphoribosyl formamidocarboxamide	8
265.04719	L-2-Aminoadipate adenylate	5
431.16524	S-Adenosylmethionine	14
274.06847	Indolelactic acid	0
	L-Aspartyl-4-phosphate	3
262.13999		
305.12324	2-Phenylethanol glucuronide	7
	Myristic acid	16
145.11067	Ethanolamine	19
	4-Guanidinobutanoic acid	20
361.12617	Deoxyguanosine	1
	N-Acetylglutamic acid	5
	scyllo-Inositol	0
263.12377	D-Galactose	0
	myo-Inositol	0
	Beta-D-Glucose	0
	D-Fructose	0
	Allose	0
	L-Sorbose	0
	D-chiro-Inositol	0
	L-Galactose	0
314.13616	Phenylacetic acid	8
	4-Hydroxyphenylacetaldehyde	8
	5-Methylcytosine	8
519.24329	Leukotriene D4	13
	D-Glucosaminide	14
808.40018		
245.11304	4-Hydroxyproline	10
	5-Aminolevulinic acid	1
	N-Acetyl-L-alanine	1
	L-Glutamic gamma-semialdehyde	1
	Dolichyl b-D-glucosyl phosphate	7
645.27642	Chitin	9
546.26158	Acetyl-N-formyl-5-methoxykynurenamine	11
	Alpha-N-Phenylacetyl-L-glutamine	11
	6-Hydroxymelatonin	11
476.16429	5,10-Methylene-THF	11
100.02789	4-Acetamido-2-aminobutanoic acid	3
	D-Alanyl-D-alanine	3

265.13204	L-Tyrosine	11
	Beta-Tyrosine	11
248.12395	Imidazole-4-acetaldehyde	19
	D-Xylose	2
	D-Ribose	2
	D-Ribulose	2
	L-Arabinose	2
	D-Xylulose	2
	L-Ribulose	2
	L-Threo-2-pentulose	2
234.10846	Guanine	6
	Deoxycytidine	8
374.15788	D-Phenyllactic acid	5
	N-Acetyl-L-glutamate 5-semialdehyde	8
165.06557	4-Pyridoxolactone	2
	Xanthurenic acid	2
	Indole-5,6-quinone	2
	Isonicotinic acid	2
	5,6-Dihydro-5-fluorouracil	5
	1-Methylnaphthalene	11
460.35634	3-Keto-4-methylzymosterol	2
467.22164		
261.15841		
175.00406		
204.95504		
809.26638		
703.51683		
1092.52266	CDP-DG (18:1(11Z)/22:5(4Z,7Z,10Z,13Z,16Z))	13
	Lucidenic acid D2	6
	Glycinoeclepin C	6
	Pisumsaponin I	11
	CDP-DG (20:3(5Z,8Z,11Z)/20:3(8Z,11Z,14Z))	13
771.31028	Cyproterone	11
	N-Methylporphyroxine	13
	NNAL-N-glucuronide	4
	Secoeremopetasitolide A	15
	Bufotenine O-glucoside	12
655.34783	PGP(i-12:0/i-12:0)	12
	Indecainide	11
	2-Hexaprenyl-3-methyl-5-hydroxy-6-methoxy-1,4-benzo-quinol	12
	1-Palmitoylglycerophosphoinositol	12
1354.66148	S-(11-hydroxy-9-deoxy-delta12-PGD2)-glutathione	10
	Nafarelin	1
	S-(9-hydroxy-PGA1)-glutathione	6

	Riboflavine 2',3',4',5'-tetrabutanoate	10
454.18202	Tryptophyl-Tryptophan	5
454.1821	Prolyl-Cysteine	6
454.18203	Glycyl-Methionine	6
	Lafutidine	11
677.33066	Chloroquine	6
	Capsanthin 5,6-epoxide	9
	Ganglioside GT2 (d18:1/18:1(11Z))	10
	Foetidissimoside B	16
	Glycylserylprolylmethionylphenylalanylvalinamide	19
631.26084	Irinotecan	15
	6-allyl-8b-Carboxy-ergoline	6
	Doxorubicin-semiquinone	1
584.26513	Phenylalanylhydroxyproline	
	Tyrosyl-Proline	19
	Adefovir Dipivoxil	10
	Morphinone	18
524.27938	CL(8:0/8:0/11:0/i-12:0)	1
	Hydroxyprolyl-Valine	20
	PIP (18:2(9Z,12Z)/20:3(8Z,11Z,14Z))	16
	Vilazodone	20
263.93638		
418.15145	Dihydrozeatin-9-N-glucoside	4
104.0453	Guanidine	12
405.15596	Propyl 2-furanacrylate	1
	3-(4-methoxyphenyl) propanoic acid	1
	3-(2-hydroxyphenyl) butanoic acid	1
	N-Acetyl-L-glutamate 5-semialdehyde	11
	N, N'-diacetylchitobiose	12
450.14039		
346.09199	N2, N2-Dimethylguanosine	1
	gamma-Glutamyl-beta-(isoxazolin-5-on-2-yl) alanine	8
	Sesaminol glucosyl-(1->2)-glucoside	18
344.09486	beta-D-Glucopyranosyl anthranilate	11
	N-Glycolylneuraminic acid	13
344.09466	N-Methoxycarbonylanonaine	1
346.09163	N2, N2-Dimethylguanosine	2
389.15885		
686.20454		
408.17429		
213.00225	Oxoglutaric acid	2
	(1R,2R)-Isocitric acid	3
	Isocitric acid	3
	Succinic anhydride	3

430.30589		
308.11798		
476.31171		
371.11374	(Z)-Resveratrol 4'-glucoside	2
	3-O-a-L-Fucopyranosyl-D-glucose	16
	Galactose-beta-1,4-xylose	16
301.09371	5-O-a-L-Arabinofuranosyl-L-arabinose	1
	1-Methylinosine	4
	Guanine	7
	2-Hydroxyadenine	7
	N5-(4-Methoxybenzyl) glutamine	8
	Diethyl methylsuccinate	11
309.12132	Argininosuccinic acid	1
	Deoxyadenosine	6
	1,7-Dimethylguanosine	6
	cycloguanil	20
434.14507	Indole-3-carboxaldehyde	14
778.32206		
422.11022		
391.17678		
278.06578	3-Methoxytyrosine	4
	4-Hydroxy-1-(3-pyridinyl)-1-butanone	4
242.08919		
288.09476	8-Acetoxypinoresinol 4-glucoside	7
422.11044		
310.11365	D-1-[(3-Carboxypropyl) amino]-1-deoxyfructose	2
	5'-Deoxyadenosine	7
407.17216	L-Tryptophan	1
	3-Hydroxymethylantipyrine	1
	Corchoionol C 9-glucoside	8
267.09571	L-Kynurenine	11
183.05127	8-Hydroxypurine	6
	L-Lysine	16
	(Z)-Resveratrol 3,4'-diglucoside	16
228.0021	2,3-Dihydro-5-methyl-1H-pyrrolizine-7-carboxaldehyde	4
	2-(2-Furanyl)-3,4,5,6-tetrahydropyridine	4
	5-Acetyl-2,3-dihydro-1H-pyrrolizine	4
	Uridine diphosphate acetylgalactosamine 4-sulfate	15
	Pyridoxal 5'-phosphate	18
	Uridine diphosphate acetylgalactosamine 4-sulfate	15
229.99913	4-Chloro-1H-indole-3-acetic acid	0
	Alendronic acid	3
205.98673	L-2,3-Dihydrodipicolinate	3

	2-Furoylglycine	3
	Miconazole	5
	DL-O-Phosphoserine	15
	Phosphoserine	15
195.0182	meso-Tartaric acid	18
197.0782	Tyrosol	19
309.1211		
422.1104		
310.1138	Deoxyadenosine	6

Supplementary Table S3

Table shows all the putative metabolites identified from the 96 significantly downregulated features in 3D cultures. The maximum mass tolerance for metabolite identification (ID) was set to 20ppm. Some features led to multiple metabolite ID, whilst others did not yield an ID (blank).

m/z value	Metabolites	PPM
292.09159	Gamma-Glutamylcysteine	16
304.06311	Thymidine 3',5'-cyclic monophosphate	5
	ADP-Mannose	12
	CDP-glucose	12
420.12572	4'-Phosphopantothenoylcysteine	5
435.13275	7-Hydroxymethyl-12-methylbenz[a]anthracene sulfate	10
	4-Phosphopantothenoylcysteine	20
244.04177	N-Acetyl-L-aspartic acid	4
436.09986	3-Hydroxybutyryl-CoA	8
172.02297	5-Fluorouridine monophosphate	14
	Indoleacetaldehyde	17
326.07329	dUMP	5
312.02923	Methylmalonyl-CoA	13
	Succinyl-CoA	12
360.17		
355.07175		
141.04057	3-Methyluridine	2
	Isopropyl apiosylglucoside	3
	O-Phosphoethanolamine	9
117.11589	Thymidine 3',5'-cyclic monophosphate	9
375.04412	p-Hydroxymandelic acid	9
	3-Hydroxymandelic acid	9
	3, 5-Dihydroxyphenylacetic acid	9
268.98724	Xylulose 5-phosphate	18
	D-Ribulose 5-phosphate	18
	2'-Deoxyinosine triphosphate	18
509.3862		
677.22869		
352.18577		
221.09066	Gentisate aldehyde	6
	3-Succinoylpyridine	6
	Pyrimidine, 2,4- bis[(trimethylsilyl)oxy]-	13
375.11202	Galactonic acid	4
	1,9-Dimethyluric acid	11
163.02829	Dihydrothymine	2
264.01408		

294.09258	L-Aspartic acid	19
176.0179	Cysteic acid	15
332.0016	Lamivudine-monophosphate	18
85.08989	BLANK	
281.27259	BLANK	
133.01197	Glyoxylic acid	17
	Malonic semialdehyde	17
	Pyruvic acid	17
	6,8-Dihydroxypurine	20
	Xanthine	20
299.28338	4-Methyl-2-methylene-1-(1methylethylidene)-cyclohexane	4
400.33534	Palmitoylcholine	9
148.98602		
321.26503		
457.07453	Homocitric acid	20
675.48531	O-decanoyl-L-carnitine	8
342.22792		
600.32856	Isoleucyl-Histidine	
	4-Hydroxyestrone	
481.35534		
183.05138	2'-Deoxyguanosine 5'-monophosphate	
	3'-AMP	
	Adenosine 2'-phosphate	
	2-hydroxy-dAMP	
	Cytidine monophosphate	
	D-Glucurono-6,3-lactone	
264.08191	D-Gal alpha 1->6D-Gal alpha 1->6D-Glucose	3
	Glycerophosphoglycerol	9
279.25708	blank	
571.16912	8-Hydroxypinoresinol 4-glucoside	18
421.02973	dUMP	8
	3-b-Galactopyranosyl glucose	13
	Glucose-1,3-mannose oligosaccharide	13
557.35961	(N-Acetylglucosaminyl)2-diphosphodolichol	11
331.08879	Hypoxanthine	6
	9-Methylxanthine	6
610.09679	4-Glutathionyl cyclophosphamide	9
	AMP	12
	Lipoyl-GMP	12
815.63896		
694.12767		
235.99125	O-Phospho-4-hydroxy-L-threonine	12
	2-Amino-3-phosphonopropionic acid	13
486.17236	Geranylgeranylcysteine	8

133.02013	4'-Phosphopantothenoylcysteine	10
	5,7-Dihydro-2-methylthieno[3,4-d] pyrimidine	17
787.6056		
222.06799	Glycerylphosphorylethanolamine	17
154.03586	Methylcysteine	13
691.51451		
145.11065	4-Guanidinobutanoic acid	19
170.00984	ATP	11
	Cysteic acid	11
631.62877		
436.09986	3-Hydroxy-2-methylpyridine-4,5-dicarboxylate	1
	3-Hydroxybutyryl-CoA	8
360.17689	Indoleacetaldehyde	
358.11	Tyramine glucuronide	
177.01502		
255.03182		
175.04606		
187.04384	Homogenistic acid	17
328.0846	OPC4-CoA	14
	Guanosine	16
375.12636	Isopropylmaleate	9
257.06511	Vanillylmandelic acid	6
157.03309	Allantoic acid	20
	Gentisate aldehyde	19
244.04177	N-Acetyl-L-aspartic acid	4
381.08831	3-Hydroxymandelic acid	15
382.07219	Arginylglutamic acid	3
	3-Indole carboxylic acid glucuronide	15
	Cytidine monophosphate	17
	Cytidine 2'-phosphate	17
284.07436		
471.08641	Cysteineglutathione disulfide	1
	Pantetheine 4'-phosphate	9
	Irisolidone 7-O-glucuronide	13
	N-Lactoyl ethanolamine phosphate	16
276.07121	6-Dimethylaminopurine	1
	N2-Succinyl-L-glutamic acid 5-semialdehyde	5
	3'-Amino-3'-deoxythimidine	16
238.11518	beta-Alanyl-L-lysine	9
257.06531	Didanosine	1
	Cystathionine sulfoxide	18
293.00906	D-Glucuronic acid 1-phosphate	6
358.0612	5'-Phosphoribosyl-N-formylglycinamidine	13
	7-Methylguanosine 5'-phosphate	15
	S-Glutaryldihydrolipoamide	16

	3-Indole carboxylic acid glucuronide	19
257.0653	Didanosine	1
406.12067	NNAL-N-glucuronide	8
	gamma-L-Glutamyl-gamma-L-glutamyl-L-methionine	20
361.28183		
216.03843		
334.09991		
280.04512	Pyridoxal/pyridoxine	5
	S-(3-Oxo-3-carboxy-n-propyl) cysteine	16
	5'-Deoxy-5-fluorocytidine	20
282.04211	N-acetyl-S-(3-oxo-3-carboxy-n-propyl) cysteine	9
	(2E)-Pentenoyl-CoA	11
	Tiglyl-CoA	11
	N-acetyl-alpha-D-galactosamine 1-phosphate	15
516.35505	LysoPE(0:0/22:1(13Z))	19
221.03409	Pyroglutamylglycine	3
	4-Hydroxy-2-oxoglutaric acid	5
	Pyruvic acid	17
242.18677		
163.02829	Dihydrothymine	2
113.99074		
396.07174	7-Methylguanosine 5'-phosphate	2
	Guanosine	14
216.17089		
244.06838		
293.00906	D-Glucuronic acid 1-phosphate	6
358.0612	5'-Phosphoribosyl-N-formylglycinamidine	13
	7-Methylguanosine 5'-phosphate	15
	S-Glutaryldihydrolipoamide	16
	3-Indole carboxylic acid glucuronide	19
257.0653	Didanosine	1
406.12067	NNAL-N-glucuronide	8
112.02527	GDP-L-fucose	7

Supplementary Table S4

Table shows top 20 upregulated pathways in GBM 3D spheroids compared to 2D cultures. Match status shows the number of putative metabolites identified in each pathway relative to the number of metabolites associated with each curated pathway. p-value, -log(p), holm p, false discovery rate (FDR) and pathway impact is shown. Significant pathways (p<0.05 and FDR≤0.05) are shown in italics.

**Pathway Name**	**Match status**	**p**	**-log(p)**	**Holm p**	**FDR**	**Impact**
Arginine and proline metabolism	*19/77*	*0.0000115*	*11.373*	*9.21E-04*	*0.0009*	*0.24*
Glycine, serine and threonine metabolism	*11/48*	*0.00011*	*9.1469*	*0.008417*	*0.0043*	*0.17*
Alanine, aspartate and glutamate metabolism	*7/24*	*0.00281*	*5.8739*	*0.21651*	*0.0056*	*0.51*
Phenylalanine metabolism	*11/45*	*0.00095*	*6.9555*	*0.074366*	*0.0254*	*0.18*
Pentose and glucuronate interconversions	11/53	0.00391	5.5453	0.29684	0.0625	0.20
Valine, leucine and isoleucine biosynthesis	7/27	0.00579	5.1509	0.43455	0.0695	0.42
Cysteine and methionine metabolism	11/56	0.00608	5.1022	0.45016	0.0695	0.36
Ascorbate and aldarate metabolism	9/45	0.01131	4.482	0.82568	0.1131	0.26
Nitrogen metabolism	9/39	0.01432	4.2462	1	0.1221	0.01
Lysine biosynthesis	7/32	0.01527	4.182	1	0.1221	0.25
Phenylalanine, tyrosine and tryptophan	6/27	0.02248	3.7951	1	0.1384	0.08
Pantothenate and CoA biosynthesis	6/27	0.02248	3.7951	1	0.1384	0.28
Citrate cycle (TCA cycle)	5/20	0.02249	3.7946	1	0.1384	0.34
Tryptophan metabolism	12/79	0.03142	3.4603	1	0.1795	0.32
Pentose phosphate pathway	6/32	0.04834	3.0295	1	0.2578	0.09
Galactose metabolism	7/41	0.05338	2.9304	1	0.2669	0.08
D-Glutamine and D-glutamate metabolism	03/22	0.05951	2.8217	1	0.2800	0.44
Histidine metabolism	7/44	0.07348	2.6107	1	0.3266	0.06
Amino sugar and nucleotide sugar metabolism	11/88	0.12046	2.1165	1	0.4905	0.13
Starch and sucrose metabolism	7/50	0.12539	2.0763	1	0.4905	0.18

Supplementary Table S5

Table shows top 20 down regulated pathways in GBM 3D spheroids compared to 2D cultures. Match status shows the number of putative metabolites identified in each pathway relative to the number of metabolites associated with each curated pathway. p-value, -log(p), holm p, false discovery rate (FDR) and pathway impact is shown. Significant pathways ($p<0.05$ and FDR≤0.05) are shown in italics.

Pathway Name	Match Status	p	-log(p)	Holm p	FDR	Impact
Purine metabolism	*11/92*	*0.0005*	*7.59*	*0.04*	*0.04*	*0.10*
Vitamin B6 metabolism	*5/32*	*0.0012*	*6.71*	*0.10*	*0.05*	*0.18*
Pantothenate and CoA biosynthesis	4/27	0.0048	5.35	0.37	0.13	0.15
Pentose and glucuronate interconversions	5/53	0.0114	4.47	0.88	0.23	0.10
Alanine, aspartate, and glutamate metabolism	3/24	0.0234	3.76	1	0.37	0.26
Ascorbate and aldarate metabolism	4/45	0.0285	3.56	1	0.38	0.12
beta-Alanine metabolism	3/28	0.0351	3.35	1	0.40	0.17
Arginine and proline metabolism	5/77	0.0487	3.02	1	0.44	0.02
Pentose phosphate pathway	3/32	0.0494	3.01	1	0.44	0.12
Propanoate metabolism	3/35	0.0618	2.78	1	0.49	0.12
Pyrimidine metabolism	4/60	0.0698	2.66	1	0.51	0.19
Valine, leucine and isoleucine dégradation	3/40	0.0851	2.46	1	0.54	0.05
Taurine and hypotaurine metabolism	2/20	0.0947	2.36	1	0.54	0.09
Citrate cycle (TCA cycle)	2/20	0.0947	2.36	1	0.54	0.15
Caffeine metabolism	2/21	0.1030	2.27	1	0.55	0.03
Glycine, serine and threonine metabolism	3/48	0.1290	2.05	1	0.64	0.00
Glyoxylate and dicarboxylate metabolism	3/50	0.1410	1.96	1	0.66	0.24
Valine, leucine and isoleucine biosynthesis	2/27	0.1561	1.86	1	0.69	0.08
Cysteine and methionine metabolism	3/56	0.1791	1.72	1	0.75	0.04
Lysine biosynthesis	2/32	0.2035	1.59	1	0.78	0.02

Supplementary Table S6

Table shows putative metabolites associated with the top 3 significantly upregulated pathways in 3D spheroid cultures.

Pathways	Metabolites
**Arginine and proline**	L-Glutamic-gamma-semialdehyde
	Glutamine
	Aspartate
	Argininosuccinic acid
	Glutamate
	N-Acetyl-L-alanine
	N-Acetyl-L-glutamate 5-semialdehyde
	L-4-Hydroxyglutamate semialdehyde
	D-4-Hydroxy-2-oxoglutarate
	Guanidoacetic acid
	4-Guanidinobutanoic acid
	N2-Succinyl-L-ornithine
	N2-Succinyl-L-glutamic acid 5-semialdehyde
	(S)-1-Pyrroline-5-carboxylate
	Gamma-glutamyl-L-putrescine
	S-Adenosylmethionine
	L-Arginine phosphate
	Homocarnosine
	2,5-Dioxopentanoate
**Serine, glycine and threonine**	Guanidinoacetate
	L-Cystathionine
	Phosphoserine
	5,10-Methylenetetrahydrofolate
	L-2-Amino-3-oxobutanoic acid
	5-Aminolevulinate
	L- methionine
	Oxidized glutathione

	S-Adenosylmethionine
	Methylglyoxal
	Hydroxy pyruvic acid
**Alanine, aspartate and glutamate**	Aspartate
	Argininosuccinic acid
	Glutamate
	(S)-1-Pyrroline-5-carboxylate
	Citrate
	Phosphoribosylamine
	α-Ketoglutaric acid

Supplementary Table S7

Table shows putative metabolites associated with the top 2 significantly down regulated pathways in 3D spheroid cultures.

Pathway	Metabolites
**Purine**	Xanthine
	Uric acid
	Phospho-ribosyl-formyl-glycineamidine
	Adenosine triphosphate
	Amino-4-imidazolecarboxyamide
	2'-Deoxyguanosine 5'-monophosphate
	Guanosine
	Adenosine monophosphate (3'-AMP)
	2'-Deoxyinosine triphosphate
	Glyoxylic acid
	N6-(1,2-Dicarboxyethyl)-AMP
**Vitamin B_6**	O-Phospho-4-hydroxy-L-threonine
	3-amino 2-oxopropyl phosphate
	Pyridoxal 5 phosphate
	Pyridoxal/pyridoxine
	4-Pyridoxate
